# *Verticillium dahliae* CFEM proteins manipulate host immunity and differentially contribute to virulence

**DOI:** 10.1186/s12915-022-01254-x

**Published:** 2022-02-23

**Authors:** Dan Wang, Dan-Dan Zhang, Jian Song, Jun-Jiao Li, Jun Wang, Ran Li, Steven J. Klosterman, Zhi-Qiang Kong, Fa-Zhuang Lin, Xiao-Feng Dai, Krishna V. Subbarao, Jie-Yin Chen

**Affiliations:** 1 The State Key Laboratory for Biology of Plant Diseases and Insect Pests, Institute of Plant Protection, Chinese Academy of Agricultural Sciences, Beijing, 100193 China; 2 United States Department of Agriculture, Agricultural Research Service, Crop Improvement and Protection Research Unit, Salinas, CA USA; 3 The Institute of Flowers, Sanming Academy of Agricultural Sciences, Shaxian, 365509 Fujian China; 4 Department of Plant Pathology, University of California, Davis, c/o U.S. Agricultural Research Station, Salinas, CA USA

**Keywords:** *Verticillium dahliae*, CFEM domain, Function divergence, Suppress immunity, Iron response

## Abstract

**Background:**

*Verticillium dahliae* is a fungal pathogen that causes a vascular wilt on many economically important crops. Common fungal extracellular membrane (CFEM) domain proteins including secreted types have been implicated in virulence, but their roles in this pathogen are still unknown.

**Results:**

Nine secreted small cysteine-rich proteins (VdSCPs) with CFEM domains were identified by bioinformatic analyses and their differential suppression of host immune responses were evaluated. Two of these proteins, VdSCP76 and VdSCP77, localized to the plant plasma membrane owing to their signal peptides and mediated broad-spectrum suppression of all immune responses induced by typical effectors. Deletion of either *VdSCP76* or *VdSCP77* significantly reduced the virulence of *V. dahliae* on cotton. Furthermore, VdSCP76 and VdSCP77 suppressed host immunity through the potential iron binding site conserved in CFEM family members, characterized by an aspartic acid residue in seven VdSCPs (Asp-type) in contrast with an asparagine residue (Asn-type) in VdSCP76 and VdSCP77. *V. dahliae* isolates carrying the Asn-type CFEM members were more virulent on cotton than those carrying the Asp-type.

**Conclusions:**

In the iron-insufficient xylem, *V. dahliae* is likely to employ the Asp-type CFEM members to chelate iron, and Asn-type CFEM members to suppress immunity, for successful colonization and propagation in host plants.

**Supplementary Information:**

The online version contains supplementary material available at 10.1186/s12915-022-01254-x.

## Background

In the classic model of plant–pathogen interactions, immunity conferred by the pathogen-associated molecular patterns (PAMPs) may be breached by the delivery of effector proteins that interfere with PAMP-triggered host immunity (PTI) that enable pathogens to infect host plants. Effector proteins therefore play important roles in infections caused by pathogenic microbes [1, 2]. Effectors generally contain signal peptides, are composed of relatively small proteins, lack transmembrane domains, are cysteine-rich, and are localized in the host plants [3–5]. In particular, small cysteine-rich proteins (SCPs) are typical apoplastic effectors, and more and more fungal SCPs have been implicated in manipulating immunity to facilitate disease development [3, 4, 6–9].

Some fungal effector proteins possess conserved motifs, such as the Crinkler (CRN), lysin motif (LysM), or common in fungal extracellular membrane (CFEM) domains that are commonly localized at their *N*- or *C*-termini [10]. CFEM domains are fungal-specific, ~60 amino-acid-long, and contain eight characteristically spaced cysteine residues with the consensus sequence PxC [A/G] x_2_Cx_8_-_12_Cx_1-3_[x/T]Dx_2-5_CxCx_9-14_Cx_3-4_Cx_15-16_ (where x is any residue with its range shown) [11]. CFEM domains are primarily found in the glycosylphosphatidylinositol (GPI) anchored cell-wall proteins, usually near their *N* termini. Many CFEM-containing effectors examined have important roles in fungal virulence [10]. For instance, adenylate cyclase-interacting protein (ACI1) from *Magnaporthe oryzae* was the first identified CFEM protein that interacts with an adenylate cyclase (MAC1), and has key roles in the formation, growth and development of appressorium and virulence [12]. Three *Aspergillus fumigatus* CFEM-containing proteins that each possess a *C*-terminal GPI anchor, affect cell-wall stability but are not implicated in haem-iron uptake and biofilm formation, or in virulence [13]. BcCFEM1, a CFEM domain-containing protein with a putative GPI-anchored site in the necrotrophic *Botrytis cinerea*, is significantly upregulated during early stages of infection of bean leaves and influences conidial production, virulence as well as stress tolerance [14]. The rice blast fungus *M. oryzae* contains the largest number of CFEM-GPCR (G-protein coupled receptor) proteins among sequenced fungi, and among these, the CFEM proteins, WISH and Pth11, have important roles in virulence [15, 16]. In addition, a systematic analysis of the evolution of CFEM-function in the genomic sequences of >100 fungi, animals, and plants, showed that CFEM is unique to fungi, and larger numbers of CFEM were present in the pathogenic than the non-pathogenic fungi [17]. Thus, CFEM-containing proteins play critically important roles in pathogenic fungi. However, no effectors containing the CFEM domain have been identified thus far in *Verticillium dahliae*.

*V. dahliae* is a soil-borne fungal pathogen that attacks a wide range of hosts causing Verticillium wilt disease on many economically important crops [18–20]. Similar to the mechanisms discovered in other phytopathogens, *V. dahliae* secretes many effectors to manipulate host immunity during infection. The avirulence gene *Ave1* encodes a SCP protein which activates *Ve1*-mediated resistance in tomato and contributes to fungal virulence [21]. The *Verticillium*-specific protein VdSCP7 is transported into plant cell nucleus, activates host both salicylic acid and jasmonic acid signaling pathways, and alters plant immunity [22]. As a pathogen with a hemibiotrophic lifestyle, *V. dahliae* also secretes effectors that suppress plant defense responses for successful infection, including cellulose-binding protein VdCBM1, isochorismatase VdISC1, and small cysteine-rich protein VdSCP41 [23–26]. Data mining of the *V. dahliae* secretome revealed that the VdLs.17 genome encodes 127 proteins designated as SCPs (proteins ≤ 400 amino acids and including ≥ 4 cysteine residues), and some of these potentially function as effectors [27]. Our recent systematic functional analysis of 123 SCPs in *V. dahliae* Vd991 from cotton revealed that three of these SCPs could induce cell death associated with host immune responses during infection [28, 29]. However, among these SCPs identified, evidence is currently lacking on those that may play a role inhibitory to host immunity.

Recently, five CFEM effectors in the maize anthracnose fungus *Colletotrichum graminicola* were shown to suppress the Bcl-2 associated X protein (BAX)-induced programmed cell death in *Nicotiana benthamiana* [30]. Interestingly, four well known CFEM proteins Csa1, Csa2, Rbt5 and Pga7 in *Candida albicans* were demonstrated to play crucial roles in the biofilm formation and virulence through haem-iron acquisition from the conserved binding site of aspartic (Asp, D) residue within the CFEM domain [31–35]. Iron is required for the survival of pathogens, and the host plant serves as an important source of iron when pathogens invade, thus many pathogens employ multiple iron uptake systems for iron predation [36–38]. Since *V. dahliae* occupies the unique niche of plant xylem [20], it is important to sequester iron from this iron-deprived environment for colonization and proliferation. Surprisingly, *V. dahliae* causes greater disease in several hosts under iron deficiency [39, 40]. Thus, these results suggest that *V. dahliae* CFEM domain-containing proteins are likely involved in iron sequestration and manipulation of immunity and virulence, but their precise roles have not been determined.

In this study, we focused on the functional analysis of CFEM domain-containing proteins in *V. dahliae*–plant interactions. We examined the CFEM members for immunity suppression and iron sequestration to determine whether *V. dahliae* differentially employs CFEM family members to promote pathogenesis.

## Results

### CFEM family members VdSCP76 and VdSCP77 display broad-spectrum cell death suppression

**Fig. 1:**
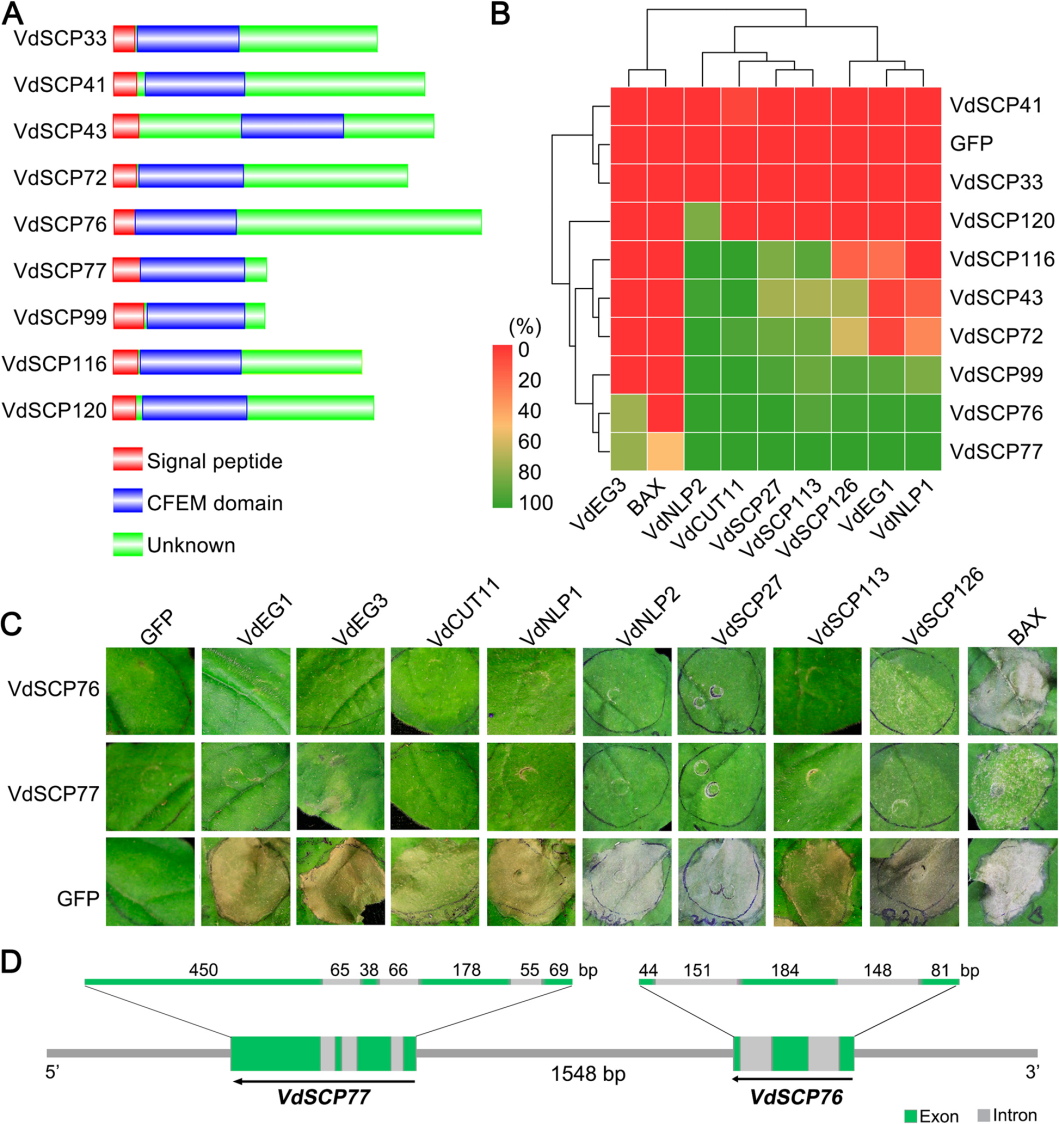
Identification of the broad-spectrum cell death suppression of small cysteine-rich CFEM protein family members in *Verticillium dahliae*. **A** Features of the primary structures of CFEM-containing SCPs. Red, blue, and green columns represent the signal peptide, CFEM domain, and unknown peptide, respectively. **B** Heat map of suppression activity of nine CFEM-containing VdSCPs against other identified *V. dahliae* cell death-inducing proteins VdSCP27, VdSCP113, VdSCP126, VdNLP1, VdNLP2, VdEG1, and VdEG3 in *Nicotiana benthamiana* leaves. The co-expression of CFEM-containing VdSCPs with Bcl-2-associated X protein (BAX) was used as control. The percentage of inhibition was the number of necrosis leaves in the total number of treated leaves. The heatmap scale bar represents the necrosis suppression ratio of nine CFEM-containing VdSCPs. **C** Suppression activity of nine CFEM-containing VdSCPs against known *V. dahliae* cell death-inducing proteins VdSCP27, VdSCP113, VdSCP126, VdNLP1, VdNLP2, VdEG1, and VdEG3 transiently expressed in 4-week-old *N. benthamiana* leaves. GFP expression was used as a control. **D** Features of the gene structures of CFEM-containing VdSCPs VdSCP76 and VdSCP77. Green and gray columns represent exon and intron regions, respectively. Arrows indicate the direction, 5′ > 3′, of *VdSCP76* and *VdSCP77*

The suppression of immunity by 120 VdSCPs in *V. dahliae* was identified by co-infiltrating the VdSCPs with a known effector VdEG1 that acts as a PAMP to induce cell death on *N. benthamiana* [24]. Of the 120 VdSCPs, transient expression of nine members (VdSCP23, VdSCP38, VdSCP58, VdSCP60, VdSCP65, VdSCP70, VdSCP76, VdSCP77 and VdSCP78, Additional file 1: Table S1) completely inhibited cell death induced by VdEG1, unlike the positive control VdEG1 that caused cell death on *N. benthamiana* leaves at 6 days after infiltration (Additional file 2: Fig. S1). Of these nine, only VdSCP76 and VdSCP77 encode CFEM domain-containing proteins (Fig. 1A; Additional file 1: Table S2). Seven other CFEM-containing VdSCPs (VdSCP33, VdSCP41, VdSCP43, VdSCP72, VdSCP99, VdSCP116 and VdSCP120) (Fig. 1; Additional file 1: Table S2), failed to suppress the cell death caused by VdEG1 fully (Additional file 2: Fig. S2).

To further examine the cell death inhibiting functions of the nine CFEM-containing VdSCPs, each was co-expressed with eight known cell death-inducing proteins (VdCUT11, VdEG1, VdEG3, VdNLP1, VdNLP2, VdSCP27, VdSCP113 and VdSCP126) and the programed cell death factor Bcl-2-associated X protein (BAX), respectively [24, 25, 29, 41, 42]. Remarkably, most of the CFEM family members (except VdSCP33 and VdSCP41) displayed a capacity to suppress cell death at varying levels when induced by the different effectors (Fig. 1B; Additional file 2: Fig. S2). VdSCP76 and VdSCP77, however, demonstrated the broadest spectrum of cell death suppression against all tested effectors on *N. benthamiana* (Fig. 1B, C). Interestingly, *VdSCP76* and *VdSCP77* are positioned adjacent (1548 bp of physical distance) to each other on chromosome 5 (Fig. 1D). Together, these results suggest that the CFEM-containing effectors are of critical importance during host–*V. dahliae* interactions, and VdSCP76 and VdSCP77 are the two CFEM members that play crucial roles in immunity manipulation with their broad-spectrum ability to suppress cell death.

### VdSCP76 and VdSCP77 localize on plant plasma membrane prior to the cell death suppression function

**Fig. 2:**
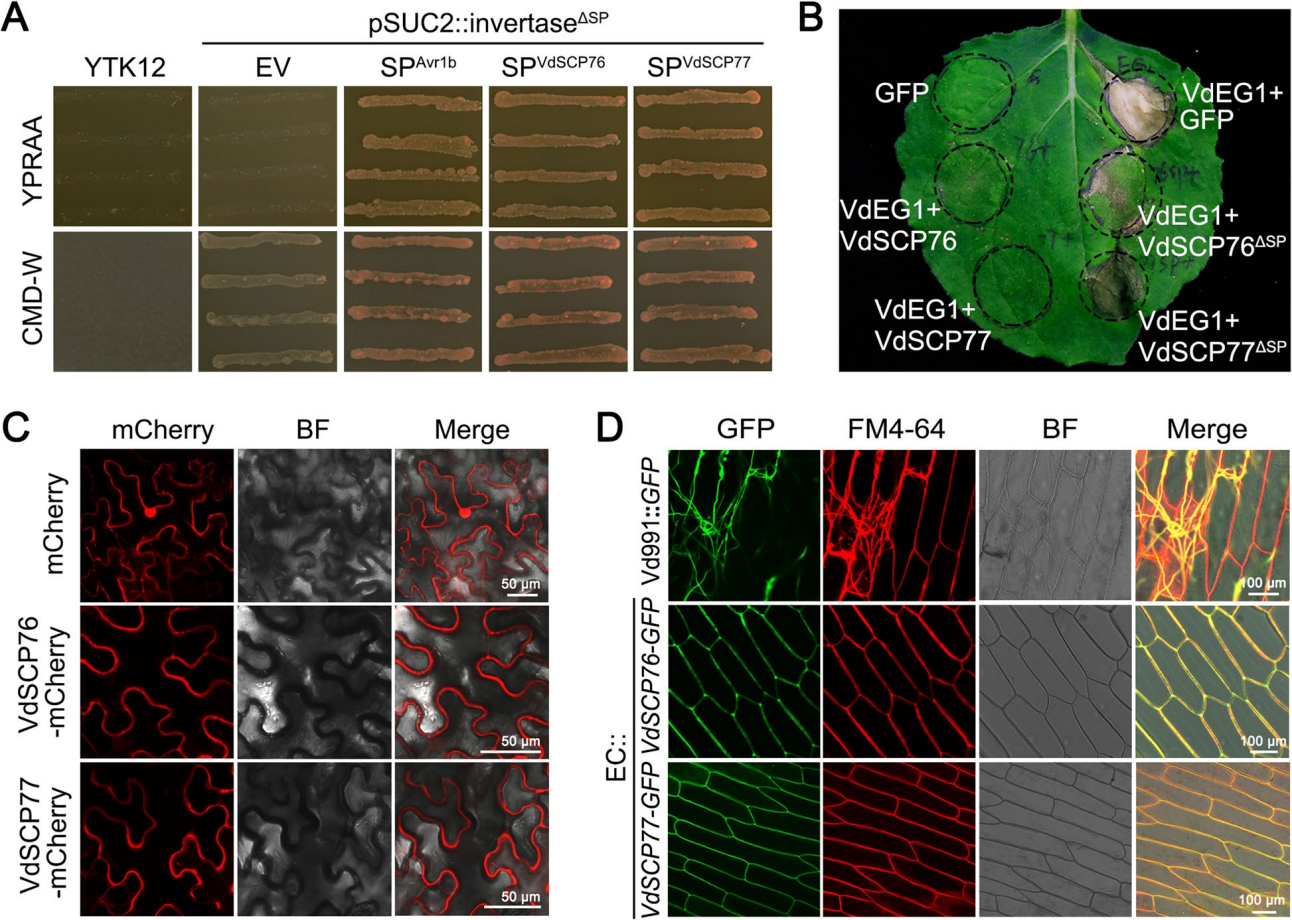
Analysis of the secretion and subcellular localization of the CFEM-containing  proteins VdSCP76 and VdSCP77 from *Verticillium dahlia*e. **A** Functional validation of the putative *N*-terminal signal peptide of VdSCP76 and VdSCP77 by a yeast signal trap assay. The sequence of the putative VdSCP76 or VdSCP77 signal peptide was fused in-frame to the invertase sequence in the vector pSUC2, and then transformed into the yeast strain YTK12. The untransformed YTK12 and the YTK12 carrying the empty pSUC2 vector were used as negative controls. The signal peptide of the oomycete effector Avr1b was used as a positive control. **B** Functional analysis of the signal peptides for the cell death suppression activities of VdSCP76 and VdSCP77 against VdEG1 in *Nicotiana benthamiana* leaves. Deletion of the signal peptide of VdSCP76 or VdSCP77 resulted in no suppression against the cell death induced by VdEG1 in 4-week-old plants at 6 days after infiltration. GFP was used as control. **C** Subcellular localization of C-terminal mCherry tagged VdSCP76 and VdSCP77 when transiently expressed in *N. benthamiana* leaves. The mCherry fluorescence was scanned by a Leica TCS SP8 confocal microscopy system with an excitation wave length of 580 nm and emission of 610 nm. Bars = 50 μm. **D** The secretion and localization analysis of VdSCP76 and VdSCP77. The conidial suspension from EC::*VdSCP76-GFP* or EC::*VdSCP77-GFP* was co-incubated with onion epidermal cells for 4 days. Wild-type Vd991-GFP was used as control. The fluorescence was scanned by a Leica TCS SP8 confocal microscopy system with an excitation wavelength of 488 nm and emission of 510 nm for GFP, and excitation of 543 nm and emission at 562 nm for FM4-64 dye, respectively. Bars = 100 μm

As typical VdSCPs, each of the nine CFEM family VdSCPs of *V. dahliae* contains a signal peptide and are secreted extracellularly (Additional file 1: Table S2). To confirm the secretory characteristics of VdSCP76 and VdSCP77, signal peptide-mediated protein secretion was detected using the yeast signal trap system [43]. The signal peptides of both VdSCP76 and VdSCP77 had the ability to mediate the secretion of invertase after fusing with the invertase gene (which lacks a signal peptide in the plasmid pSUC2) in yeast, and successfully conferred the ability of yeast strain YTK12 to utilize raffinose as the sole carbon source and grow normally (Fig. 2A), similar to the signal peptide from Avr1b used as a positive control. This suggested that VdSCP76 and VdSCP77 were most likely secreted to the extracellular space under the guidance of their signal peptides. Furthermore, the immunity suppression ability was observed by co-infiltration of signal peptide deletion mutants from VdSCP76 and VdSCP77 (VdSCP76^ΔSP^ and VdSCP77^ΔSP^) with VdEG1. Both VdSCP76 and VdSCP77 reduced the ability to suppress cell death after deletion of the signal peptide, compared to the intact native genes that completely suppressed cell death induced by VdEG1 (Fig. 2B). VdEG1 was successfully expressed in *N. benthamiana* with all infiltrations as revealed by the immunoblot analysis (Additional file 2: Fig. S3A). These results indicated that VdSCP76 or VdSCP77 must be transported to extracellular space for them to exhibit cell death suppression.

To further detect the subcellular location of VdSCP76 and VdSCP77 in host cells, the native VdSCP76 and VdSCP77 sequences fused with mCherry fluorescence protein sequence (VdSCP76/77-mCherry) was transiently expressed in *N. benthamiana* leaves. The red fluorescence signals of VdSCP76 and VdSCP77 mainly localized around the periphery of *N. benthamiana* cells, especially on the plasma membrane (Fig. 2C; Additional file 2: Fig. S3B). Furthermore, during *V. dahliae*–host interactions, the locations of VdSCP76 and VdSCP77 in host cells were examined by fusing each with the green fluorescent protein (GFP). The *VdSCP76* (Δ*VdSCP76*) and *VdSCP77* (Δ*VdSCP77*) deletion strains were first constructed by replacing the gene sequence with a hygromycin resistance cassette through homologous recombination (Additional file 2: Fig. S4), and the native *VdSCP76* and *VdSCP77* sequences fused with *GFP* were then re-introduced into Δ*VdSCP76* and Δ*VdSCP77* strains (EC::*VdSCP76-GFP* and EC::*VdSCP77-GFP*), respectively (Additional file 2: Fig. S5C and S5D). Subsequently, the positive transformants were incubated with onion epidermal cells to investigate the green fluorescence signal distribution. The results clearly showed that the green fluorescence signals of VdSCP76 or VdSCP77 localized around the onion cell periphery, and the signals were more concentrated on the plasma membrane and overlapped with the red fluorescence signal of cell membrane fluorescence dye FM4-64 (Fig. 2D; Additional file 2: Fig. S3C), unlike the negative *V. dahliae* control that carried only the GFP. There were rare GFP signals extracellularly around the onion cells but most signals were concentrated in the mycelium of *V. dahliae* and not secreted (Fig. 2C). These results further confirmed that VdSCP76 and VdSCP77 localized at the host cell periphery, especially at the plasma membrane following secretion from *V. dahliae*.

### VdSCP76 and VdSCP77 significantly enhanced host susceptibility by suppressing immunity

**Fig. 3:**
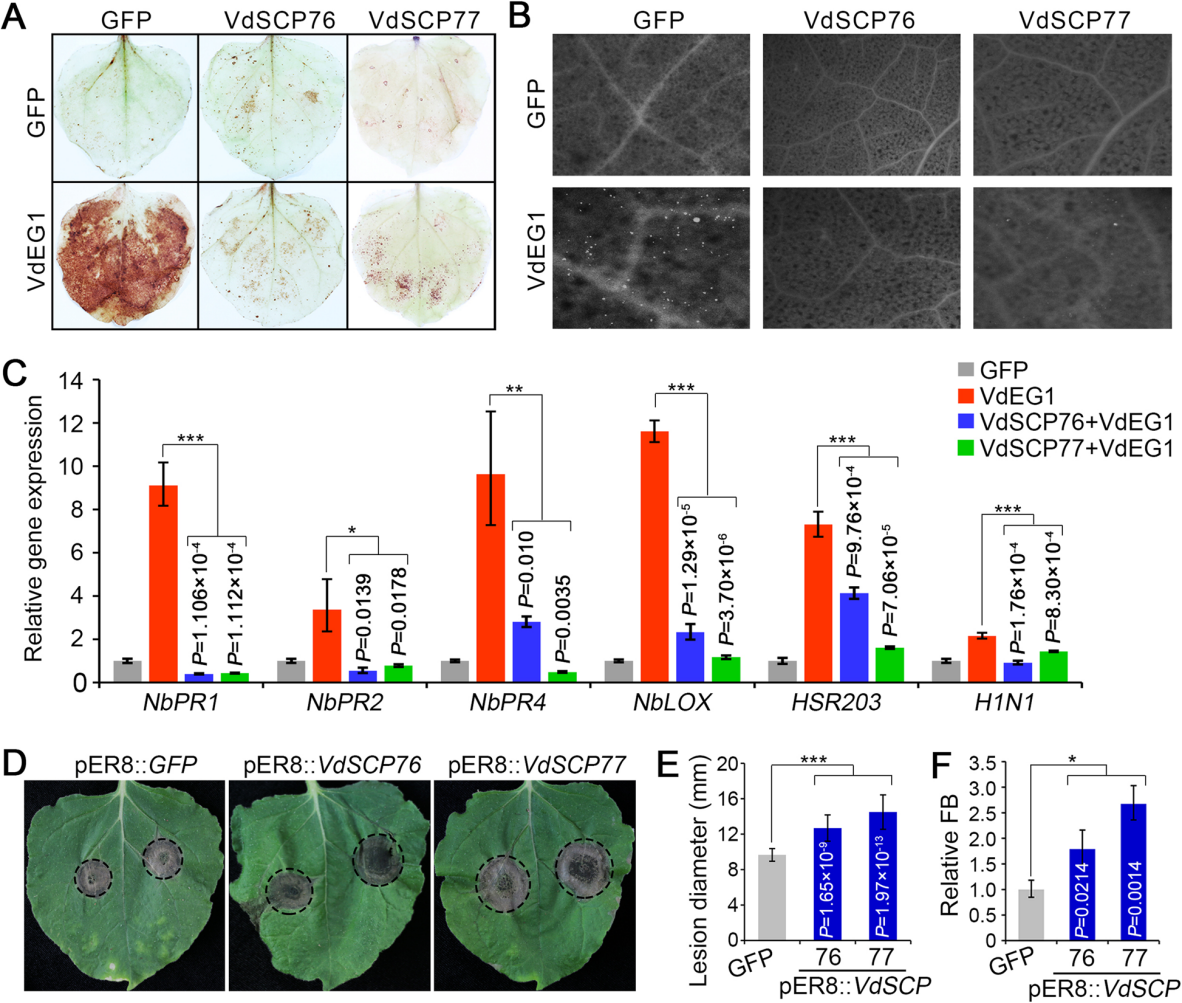
*Verticillium dahliae* proteins VdSCP76 and VdSCP77 enhance host susceptibility by suppressing immunity. **A, B** ROS accumulation and callose deposition after transient co-expression of VdSCP76 or VdSCP77 with VdEG1 in *Nicotiana benthamiana* leaves from 4-week-old plants were determined by 3,3'-diaminobenzidine (DAB) and aniline blue staining, respectively. **C** Expression of resistance-related gene was detected using reverse transcription quantitative PCR at 2 days after co-agro-infiltration of VdSCP76 or VdSCP77 with VdEG1 in *N. benthamiana* leaves. In all above, single infiltrations of VdEG1 and GFP were used as the positive and negative controls, respectively. **D** Disease symptoms of *Botrytis cinerea* on *N. benthamiana* plants overexpressing VdSCP76 and VdSCP77. *N. benthamiana* leaves from a 5-week-old plants were inoculated with 10 μL of 1 × 10^7^ conidia/ml *B. cinerea*, and symptoms analyzed at 4 days after inoculation. **E,F** Lesion development of *B. cinerea* on *N. benthamiana* leaves was evaluated from 4 days post-inoculation by determining the lesion diameter on leaves from six plants each. Fungal biomass of *B. cinerea* was determined by quantitative PCR. Error bars represent standard errors. *, **, and *** represent significant differences at *P* < 0.05, *P* < 0.01, and *P* < 0.001, respectively, between positive control VdEG1 and co-expression of VdEG1 with VdSCP76 or VdSCP77, according to one-way analysis of variance (ANOVA)

The immunity triggered by PAMPs can restrict pathogen infection on host plants, and PAMP-triggered immune responses include reactive oxygen species (ROS) burst, callose deposition, and induction of defense-related genes [44, 45]. VdSCP76 and VdSCP77 displayed the ability to suppress cell death induced by typical PAMPs and also other effectors (Fig. 1B, C; Additional file 2: Fig. S2). To confirm the suppression of immunity by VdSCP76 and VdSCP77, they were individually co-expressed transiently with the typical PAMP VdEG1 in *N. benthamiana*. As expected, the ROS accumulation and callose deposition triggered by VdEG1 were significantly suppressed by VdSCP76 and VdSCP77 (Fig. 3A, B; Additional file 2: Fig. S6A and S6B), and the electrolyte leakage caused by VdEG1 was also significantly alleviated following co-expression with VdSCP76 or VdSCP77 (Additional file 2: Fig. S6C). Correspondingly, the defense-related genes (*NbPR1*, *NbPR2*, *NbPR4*, *NbLOX*, *HSR203*, *HIN1*) induced by VdEG1 were significantly suppressed when co-expressed with VdSCP76 or VdSCP77 in *N. benthamiana* leaves (Fig. 3C).

To further investigate the role of VdSCP76 and VdSCP77 in enabling plant infection by interfering with host immunity, susceptibility of *N. benthamiana* to *Botrytis cinerea* was examined. Transgenic lines of *N. benthamiana* with *VdSCP76* and *VdSCP77* (pER8::*VdSCP76* and pER8::*VdSCP77*) were generated by the estrogen-inducible XVE system (LexA-VP16-Estragon Receptor) that induces gene (*VdSCP76* or *VdSCP77*) expression and a rapid accumulation of protein with the estrogen treatment [46]. Compared to the control pER8::*GFP* transgenic line, the lesion size on transgenic *N. benthamiana* leaves that rapidly accumulated VdSCP76 and VdSCP77 proteins with an estrogen treatment was higher and the fungal biomass was significantly increased 4 days after inoculation with *B. cinerea* (Fig. 3D–F). These results further confirmed that VdSCP76 and VdSCP77 have the ability to suppress immunity and increase plant susceptibility to facilitate pathogen colonization.

### Cysteine residues in CFEM domain of VdSCP76 and VdSCP77 are essential for immunity suppression

**Fig. 4:**
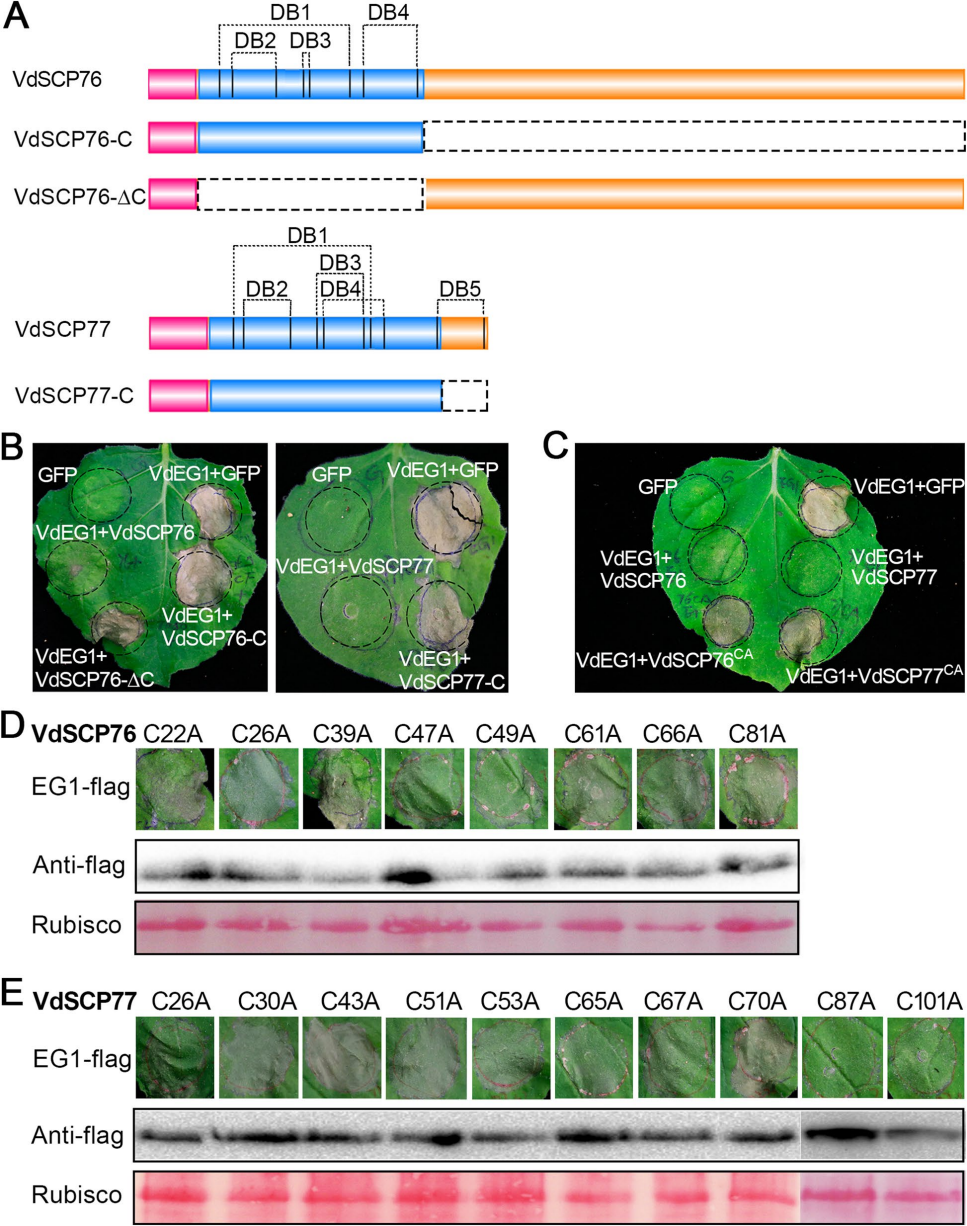
Cysteine residues in the CFEM domains of VdSCP76 and VdSCP77 are essential for immunity suppression. **A** The primary structures and putative disulfide bonds (DBs) of VdSCP76 and VdSCP77. Pink, blue, and orange blocks indicated as signal peptide, CFEM domain, and unidentified sequence. Vertical strings represented cysteine residues sites position. **B, C** The cell death suppression activity of the wild-type, truncated proteins, and cysteine residue site-directed mutant proteins of VdSCP76 and VdSCP77 were determined by transient co-expression with VdEG1, respectively, in 4-week-old *Nicotiana benthamiana* leaves. “CA” indicates that all cysteine residues were replaced with alanine. GFP and VdEG1 were used as controls. **D, E** The function of single cysteine residues in VdSCP76 and VdSCP77 were detected by cell death inhibition against VdEG1 assays in 4-week-old *N. benthamiana* leaves. “C-No.-A” represents the single cysteine residue replaced with alanine in the respective position in VdSCP76 and VdSCP77. The efficiency of transient expression of the cell death-inducing gene VdEG1 was validated by western blotting with FLAG tag antibody and horseradish peroxidase (HRP)-linked immunoassay. Ponceau S-stained Rubisco protein is shown as a total protein loading control

Structural analyses of VdSCP76 and VdSCP77 revealed that each possesses a CFEM domain (Fig. 4A), and compared to VdSCP77, there is an additional amino acid sequence extension at the *C*-terminus of VdSCP76 (Fig. 4A). To investigate the association of sequence characteristics with suppression of immunity, the immunity suppression activities were examined using truncated VdSCP76 and VdSCP77 proteins. The results showed that transient expression of the truncated protein VdSCP76-C or VdSCP77-C (VdSCP76 or VdSCP77 that only retained the signal peptide sequence and CFEM domain) lost the ability to suppress cell death induced by VdEG1 at 6 days after co-infiltration (Fig. 4B). Moreover, the deletion of the CFEM domain in VdSCP76 (VdSCP76-*∆*C) also resulted in the loss of cell death-suppressing function on *N. benthamiana* induced by VdEG1 (Fig. 4B). The successful expression of VdEG1 in the above treatments was verified by immunoblotting (Additional file 2: Fig. S7A). Together, these results suggested that the retention of structural and sequence integrity of VdSCP76 and VdSCP77 are important for their roles in cell death suppression.

The CFEM domain is a cysteine-rich motif that typically contains eight cysteine residues [11]. Sequence analysis showed that VdSCP76 contains eight cysteine residues and VdSCP77 contains ten that form four and five potential disulfide bonds, respectively, based on the prediction results from DiANNA 1.1 web server [47] (Fig. 4A). To further confirm the importance of potential disulfide bonds formed by cysteine residues in the suppression of immunity, we employed the site-direct mutagenesis (alanine substitute for cysteine, C >> A) to assess this function. The results revealed that transient expression of the variant in either VdSCP76 or VdSCP77 displayed the loss of cell death suppression function induced by VdEG1 on *N. benthamiana* (Fig. 4C). The mutants with any one of the cysteine residues substituted (C >> A) in the CFEM domain of VdSCP76 or VdSCP77 led to the loss of suppression of cell death induced by VdEG1 (Fig. 4D, E). However, VdSCP77 contains an additional predicted disulfide bond that is formed by two cysteine residues, one located in the CFEM domain and another in the extended region of the *C*-terminus (Fig. 4A). Site-directed mutagenesis of these two cysteine residues alone did not affect the immunity suppression function of VdSCP77 (Fig. 4E). Immunoblotting analysis confirmed that VdEG1 was expressed correctly in all above treatments (Fig. 4D, E and Additional file 2: Fig. S7B). Together, these results suggested that the eight cysteine residues likely associated with disulfide bonds in the CFEM domains are important for immunity suppression by VdSCP76 and VdSCP77.

### VdSCP76 and VdSCP77 play crucial roles in virulence

**Fig. 5:**
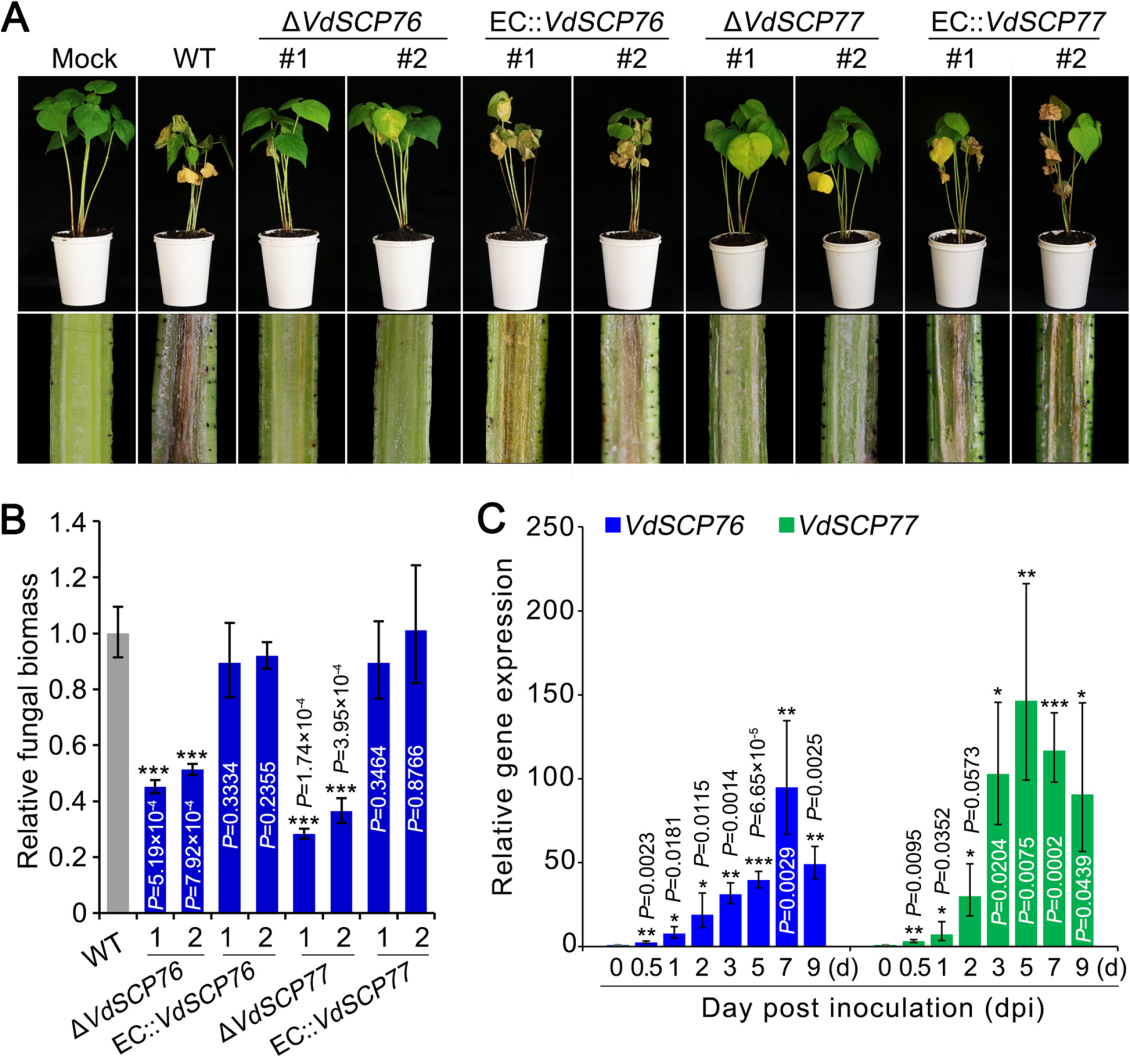
Virulence assays with *VdSCP76* and *VdSCP77* deletion mutant and complemented transformant strains of *Verticillium dahliae* on cotton. **A** Phenotypes of cotton seedlings inoculated with *VdSCP76* and *VdSCP77* gene deletion mutants and the complemented transformants. Three-week-old cotton (cv. Junmian 1) seedlings were inoculated with wild-type *V. dahliae*, *VdSCP76*, and *VdSCP77* gene deletion mutants and complemented transformants, and the disease symptoms were observed at 3 weeks post-inoculation (wpi). The phenotypes of cotton are shown at the top and the discoloration of shoot longitudinal sections is shown at bottom. **B** The fungal biomass of *V. dahliae* in cotton was determined by quantitative PCR. **C** Expression of *VdSCP76* and *VdSCP77* during infection of cotton roots. Three-week-old cotton plants (cv. Junmian 1) were inoculated with wild-type *V. dahliae* Vd991 and the roots were harvested at different time points after inoculation. Reverse transcription-quantitative PCR was performed to determine the expression levels of *VdSCP76* and *VdSCP77* relative to *V. dahliae EF-1α*. Error bars represent standard errors. *, **, and *** indicate statistical significance at *P*<0.05, *P*<0.01, and *P*<0.001, respectively, in controls and different treatments (between WT and each knockout mutant or complementary transformant, or between 0 dpi and each time point of infection) according to one-way analysis of variance (ANOVA)

The roles of VdSCP76 and VdSCP77 in suppressing immunity and enhancing virulence were further investigated. Two independent deletion strains for each gene were selected for inoculation of cotton plants using the root-dip method [24]. The results showed that deletion of either *VdSCP76* or *VdSCP77* (*∆VdSCP76* and *∆VdSCP77*) significantly impaired virulence on cotton plants at 21 days post-inoculation, compared to the leaf necrosis and wilting observed following inoculation with the wild-type strain Vd991 (Fig. 5A). The virulence of the complemented transformants, in which the wild-type *VdSCP76* or *VdSCP77* gene was re-introduced into the respective deletion mutants (EC::*VdSCP76* and EC::*VdSCP77*), was comparable to that of the wild-type strain Vd991 (Fig. 5A). Fungal biomass analyzed by quantitative PCR (qPCR) in inoculated cotton plants revealed that deletion of *VdSCP76* or *VdSCP77* resulted in reduced fungal biomass as compared to the fungal biomass of the wild-type strain and in plants inoculated with the complementary transformants (Fig. 5B). Investigation of the transcript levels of *VdSCP76* and *VdSCP77* revealed that both were significantly upregulated during infection of cotton plants (0.5–9 days post-inoculation) (Fig. 5C), confirming that *VdSCP76* and *VdSCP77* play important roles during host infection. Furthermore, the effects of deletion of both *VdSCP76* and *VdSCP77* on virulence were investigated by examining the inoculated cotton plants. Deletion of both *VdSCP76* and *VdSCP77* significantly reduced the strain virulence on cotton, while the virulence was restored in the complementation mutants (Additional file 2: Fig. S8). These results confirmed that the immunity suppressors VdSCP76 and VdSCP77 play critical roles in virulence of *V. dahliae* on cotton.

### *Verticillium dahliae* CFEM-containing VdSCPs differentially contribute to virulence

**Fig. 6:**
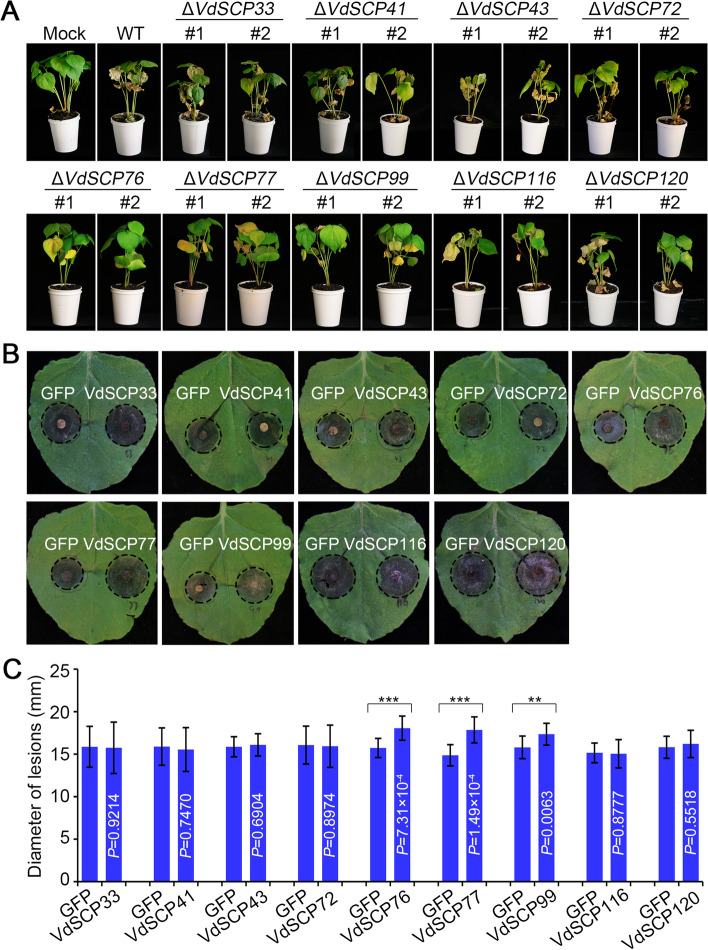
Functional differentiation of the CFEM-containing VdSCPs in *Verticillium dahliae*. **A** Virulence assays with CFEM-containing VdSCPs gene deletion mutant strains of *V. dahliae* on cotton. Three-week-old cotton seedlings were inoculated with wild-type *V. dahliae* and gene deletion mutant strains, and the disease symptoms were observed at 3 weeks after inoculation. **B** *Botrytis cinerea* infection assays in the 4-week-old *Nicotiana benthamiana* leaves that were firstly transient expression with *Agrobacterium tumefaciens* Gv3101 carrying the vector harboring GFP as control on the left of leaves and carrying the recombinant vector harboring CFEM-containing VdSCPs on the right of leaves. The phenotypes (**B**) and diameters (**C**) of lesions were examined at 4 days after inoculation with *B. cinerea*. Error bars represent standard errors. ** and *** represent statistical significance at *P* < 0.01 and *P* < 0.001, respectively, between the different VdSCPs and GFP control according to one-way analysis of variance (ANOVA)

Results above showed that nine CFEM-containing VdSCPs displayed differential suppression of cell death induced by the different types of effectors (Fig. 1B; Additional file 2: Fig S2), with VdSCP76 and VdSCP77 having the broadest-spectrum cell death suppression compared to the other seven members (Fig. 1B, C; Additional file 2: Fig. S2). These results indicated that their roles during infection are likely to be different, and therefore, the virulence of deletion mutants of each was further investigated. Two independent positive deletion strains for each of the nine genes were selected for virulence assays on cotton. Except for the deletion mutants from *VdSCP76*, *VdSCP77*, and *VdSCP99* showing significantly impaired virulence at 21 days post-inoculation, the other six deletion mutants showed no obvious change in virulence relative to the wild-type strain Vd991 (Fig. 6A; Additional file 2: Fig S9A). Furthermore, quantification of the fungal biomass in cotton stems confirmed that colonization by Δ*VdSCP76,* Δ*VdSCP77*, and Δ*VdSCP99* was significantly reduced relative to the other six strains (Additional file 2: Fig. S9B). Furthermore, the development of *B. cinerea* infection was significantly expanded in *N. benthamiana* leaves in which *VdSCP76*, *VdSCP77*, and *VdSCP99* were transiently expressed, but the other six members, when transiently expressed, yielded lesion sizes similar to the GFP control (Fig. 6B). The *B. cinerea* lesion diameters and biomass were also consistent with above phenotype, with only *VdSCP76*, *VdSCP77*, and *VdSCP99* showing differences from the GFP control (Fig. 6C; Additional file 2: Fig. S9C). The induced expression levels of *VdSCP76* and *VdSCP77* were over 100-fold higher 5 days after inoculation and were over 200-fold higher for *VdSCP77* 7 days after inoculation (Additional file 2: Fig. S10). The expression of other genes also showed varying degrees of upregulation (Additional file 2: Fig. S10)*.* These results strongly suggested that the nine CFEM-containing VdSCPs displayed differences in virulence depending on their ability to suppress immunity, with *VdSCP76* and *VdSCP77* playing the most significant roles in virulence.

### VdSCP76 and VdSCP77 suppress host immunity through the potential iron binding site conserved in the CFEM family

**Fig. 7:**
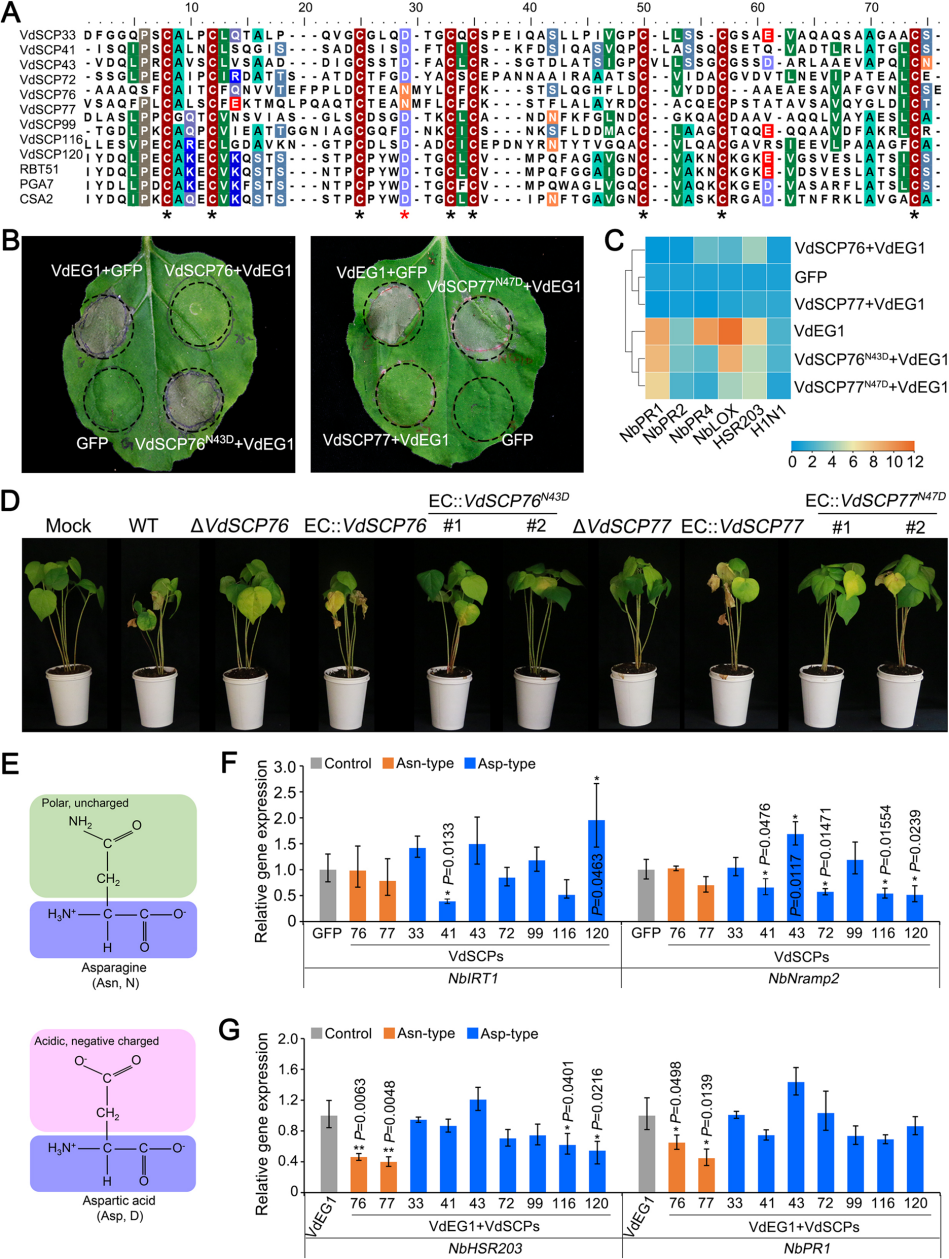
Functional analyses of conserved asparagine residues in the CFEM domain-containing proteins VdSCP76 and VdSCP77 from *Verticillium dahliae*. **A** Sequence alignment of the CFEM domain from CFEM-containing VdSCPs in *V. dahliae* and heme-iron acquisition related CFEM proteins in *Candida albicans*. The sequences were aligned using Clustal W2 [48]. Black and red asterisks represent the position of the eight conserved cysteine residues and the mutation of the conserved site. **B** Cell death suppression activity of native and D>N site-directed mutant proteins of VdSCP76 and VdSCP77 were detected by co-expressing transiently with VdEG1 in 4-week-old *Nicotiana benthamiana* leaves. GFP and VdEG1 were used as controls. **C** Heat map of resistance-related genes expression in *N. benthamiana* leaves infiltrated with wild-type and asparagine site-mutated proteins of VdSCP76 and VdSCP77 with co-expression of the cell death inducer VdEG1. Gene expression was detected by reverse transcription-quantitative PCR (RT-qPCR) at 2 days after agro-infiltration. **D** Virulence assays of wild-type Vd991, *VdSCP76*, and *VdSCP77* gene deletion mutant strains, native *VdSCP76* and *VdSCP77* complement and asparagine site-mutated complement transformants of *V. dahliae* on cotton (cv. Junmian 1). Three-week-old seedlings of cotton plants were inoculated, and Verticillium wilt symptoms were photographed at 3 weeks post-inoculation (wpi). **E** Molecular structural formulas of asparagine and aspartic acid. **F** CFEM-containing VdSCPs (Asn-type and Asp-type) were expressed transiently in 4-week-old *N. benthamiana* and the leaves were collected at 2 days after agro-infiltration. The iron transport-associated gene (*NbIRT1*: iron-regulated transporter 1; *NbNramp2*: natural resistance-associated macrophage protein 2) expression levels were detected by RT-qPCR. GFP was served as control. **G** Expression of genes related to the immune response of *N. benthamiana* was determined by RT-qPCR. The CFEM-containing VdSCPs were co-expressed with VdEG1 transiently, and single infiltration of VdEG1 was used as control. Error bars represent standard errors. * and ** represent significant differences at *P* < 0.05 and *P* < 0.01, respectively, between the different VdSCPs treatments and GFP control according to one-way analysis of variance (ANOVA)

Previous studies have shown that *Candida albicans* employs the CFEM protein to extract heme-iron from hemoglobin to cope with extremely low free-iron concentrations in the host tissues, and this function is dependent upon an aspartic acid (Asp, D) residue within CFEM domain [34]. Interestingly, while sequence alignment analysis revealed that the Asp residue was conserved among seven CFEM in *V. dahliae*, this residue diverged to an asparagine (Asn, N) in both VdSCP76 and VdSCP77 (Fig. 7A). Because of the broadest immunity suppression by VdSCP76 and VdSCP77 relative to the other CFEM-containing VdSCP members in this family, we examined the relationship between the site and suppression of immunity. We found that if the Asn residue was substituted with the Asp residue in VdSCP76 and VdSCP77, the mutant protein VdSCP76^N43D^ and VdSCP77^N47D^ completely lost the ability to suppress cell death induced by VdEG1 in *N. benthamiana* 6 days after co-infiltration (Fig. 7B). Compared with the wild-type VdSCP76 or VdSCP77, the defense-related genes induced by VdEG1 were not inhibited when treated with the mutant protein VdSCP76^N43D^ and VdSCP77^N47D^ (Fig. 7C; Additional file 2: Fig. S11). These results indicate that the site mutation in VdSCP76 and VdSCP77 compared to the other seven CFEM-containing VdSCPs was necessary for their immunity suppression function. Furthermore, the complemented strains EC::*VdSCP76*^*N43D*^ and EC::*VdSCP77*^*N47D*^ with Asn substituted to Asp failed to recover the virulence on cotton, compared to the complemented strains EC::*VdSCP76* and EC::*VdSCP77* with virulence similar to the wild-type strain (Fig. 7D). Fungal biomass and disease index measurements in cotton further supported these observations (Additional file 2: Fig. S12). These results indicated that the mutation of the potential iron binding site (D>>N) in VdSCP76 or VdSCP77 compared to other CFEM-containing VdSCPs likely caused a deficiency in iron sequestration. Thus, the potential iron binding site likely functions in suppressing immunity and affects virulence under the low iron environment in the plant xylem. These divergent functions are supported by the Asp residue in CFEM domain (Asp-type) with the negatively charged side chain binding to the positively charged iron, but the mutation of Asn residue in CFEM domain (Asn-type) probably led to the loss of the ability to bind iron due to its polar uncharged side chain (Fig. 7E).

Iron homeostasis plays a critical role in disturbing the plant immunity during host-pathogen interactions [37]. We therefore further investigated the expression of iron homeostasis-related genes in *N. benthamina* in response to the transiently expressed members of the CFEM gene family from *V. dahliae*. The *N. benthamina* genes examined included the homolog of iron-regulated transporter 1 (*NbIRT1*) and the natural resistance-associated macrophage protein 2 (*NbNramp2*), which are the central iron-uptake transporters that are responsible for the normal iron homeostasis [49, 50]. Indeed, the transiently expressed Asn-type CFEM proteins did not interfere with the expression of *NbIRT1* and *NbNramp2*, but most members of Asp-type significantly interfered with the expression of *NbIRT1* and *NbNramp2* at 48 h after transient expression in *N. benthamina* leaves (Fig. 7F). Interestingly, the expression of several defense response genes induced by VdEG1 were significantly suppressed when the Asn-type VdSCP76 and VdSCP77 CFEM proteins were transiently expressed but not as strongly or rarely for the Asp-type CFEM members (Fig. 7G). Not all the Asp-type CFEM-containing VdSCPs are involved in the regulation of iron homeostasis- or defense response-related genes (Fig. 7F, G), and this may be due to division roles of Asp-type CFEM-containing VdSCPs in the potential role of iron predation. However, the Asn residue in the iron-chelating site is necessary but not sufficient for immunity suppression, likely because the Asp-type CFEM members cannot fully gain the function of immunity suppression after mutation to the Asn-type protein (Additional file 2: Fig. S13).

**Fig. 8:**
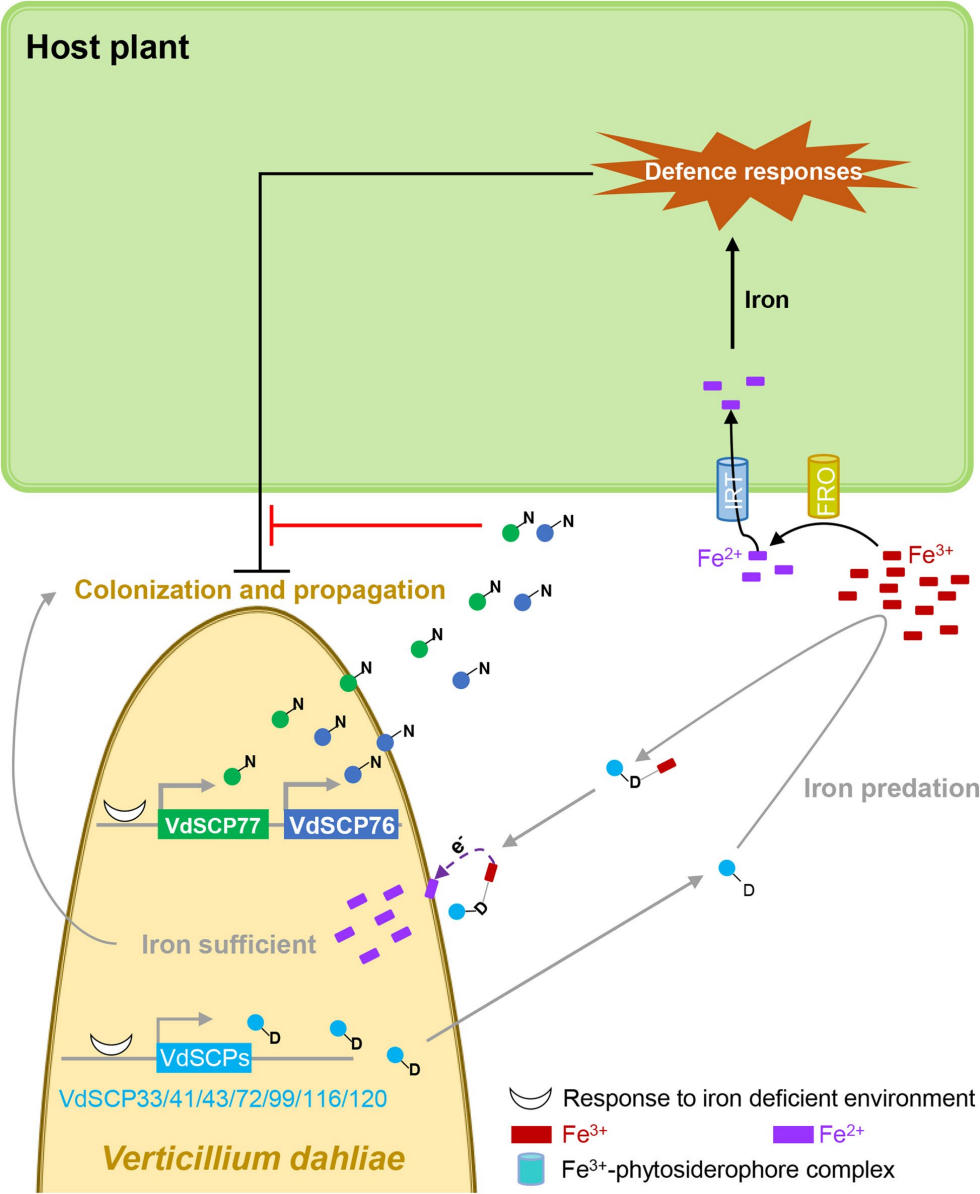
Hypothetical model for the functional differentiation of Asn-type and Asp-type CFEM-containing VdSCPs from *Verticillium dahliae*. Under the iron-insufficient environment of xylem, on the one hand, *V. dahliae* employs the Asp-type CFEM members (VdSCP33, VdSCP41, VdSCP43, VdSCP72, VdSCP99, VdSCP116, VdSCP120) to sequester iron that further aggravates the iron deficiency in the xylem and reduces host resistance; on the other hand, *V. dahliae* employs Asn-type CFEM members (VdSCP76, VdSCP77) to suppress immunity directly and to promote successful colonization. FRO: ferric-chelate reductase. IRT: iron-regulated transporter. N and D represent asparagine (Asn) type and aspartic acid (Asp) type CFEM-containing VdSCPs from *V. dahliae*, respectively

Finally, we investigated the stress response of all CFEM-containing VdSCPs under iron deficient (exogenous additive of 150 μM bathophenanthrolinedisulfonic acid disodium salt, BPS) and iron sufficient (exogenous additive of 50 μM FeCl_3_) conditions. Compared to the iron-sufficient environment, the expression of almost all CFEM-containing VdSCPs were differentially induced under iron deficiency, though those with the Asn-type displayed significant sensitivity to the iron deficiency (Additional file 2: Fig. S14). Coincidentally, the expression levels of Asp-type CFEM genes were relatively lower during infection of host plants than Asn-type CFEM members (Fig. 5C; Additional file 2: Fig. S10), which further suggested that the expression levels of Asn-type CFEM members are more sensitive than Asp-type CFEM members under the iron-insufficient environment in the host. Thus, under conditions of iron insufficiency in the xylem, *V. dahliae* may employ the Asp-type CFEM members to sequester the iron that further aggravates the iron deficiency in the xylem; meanwhile, *V. dahliae* likely employs Asn-type CFEM members under conditions of iron deficiency to suppress immunity and to promote successful colonization (Fig. 8).

## Discussion

Many fungal proteins containing CFEM domain play important roles in pathogen virulence during host–pathogen interactions [11], and some have a direct role in iron acquisition through the conserved axial ligand binding side of Asp residue in the CEFM domain [34]. In this study, nine CFEM-containing VdSCPs displayed functional differentiation in virulence and in their ability to suppress immunity. Only VdSCP76 and VdSCP77 showed broad-spectrum immunity suppression function and played critical roles in virulence. The conserved Asp residue in CFEM domain proteins were substituted by an Asn residue inVdSCP76 or VdSCP77, which suppressed the ability to respond to iron. Thus, *V. dahliae* may employ the CFEM-containing VdSCPs that contribute to virulence by their functionally divergent roles in suppressing immunity (Asn-type CFEM members) or in sequestering iron (Asp-type CFEM members) to promote successful colonization (Fig. 8).

Many fungal effectors are VdSCPs that manipulate host immunity, and those effectors involved in the suppression of host immunity directly contribute to the virulence [4]. CFEM proteins contain eight characteristically spaced cysteine residues and are widespread among fungi, and the majority studied to date are involved in host-pathogen interactions and virulence [11, 17, 51]. However, an analysis of the CFEM proteins in a few filamentous fungi, including *A. fumigatus*, *B. cinerea*, and *M. oryzae*, suggested they are mainly involved in fungal cell wall integrity, conidial production, infection structure formation, virulence, and in inducing chlorosis in *N. benthamiana* [13–16]. In this study, *V. dahliae* encoded nine CFEM-containing VdSCPs with secretory characteristics, and all except VdSCP33 and VdSCP41 differentially suppressed immunity induced by effectors and three members were clearly involved in virulence of cotton (Figs. 1 and 6). Thus, our study further confirmed that some CFEM family members in *V. dahliae* act as effectors that play a critical role in pathogen virulence.

CFEM proteins mainly locate onto the outer layer of the cell membrane, as demonstrated with Pth11 and WISH in *M. oryzae* [15, 16, 52]. The nine CFEM-containing VdSCPs in *V. dahliae* each contained a signal peptide and their subcellular localization was extracellular as predicted (Fig. 1; Additional file 1: Table S1 and S2). In *C. graminicola*, the CFEM family suppressed the BAX-induced programmed cell death in *N. benthamiana* [30]. Unexpectedly, the CFEM-containing VdSCPs in *V. dahliae* were unable to suppress cell death induced by the intracellular effector BAX absolutely (Fig. 1; Additional file 2: Fig. S2), suggesting that VdSCPs likely function in the apoplastic space during host-*V. dahliae* interactions. Furthermore, there exists a functional difference between wild-type VdSCP76 and VdSCP77 and those lacking a signal peptide (Fig. 2). These results suggested that these CFEM-containing VdSCPs act as apoplastic suppressors to manipulate immunity, especially PTI, during host-*V. dahliae* interactions.

The CFEM motif has eight conserved cysteine residues [11], which are involved in the formation of disulfide bonds that result in the stabilization of proteins. Disulfide bonds that are important for protein function have been identified in several effectors, including Avr2, Avr4, and Avr9 from *Cladosporium fulvum* [53–55] and SsSSVP1 from *Sclerotinia sclerotiorum* [56]. However, the role of eight conserved cysteine residues in the various functions of CFEM proteins was not identified. We confirmed that the eight cysteine residues in each of the CFEM domains formed four disulfide bonds in VdSCP76 and VdSCP77 and were necessary for suppression of host immunity. Any substitutions to these destroyed their immunity suppression function (Fig. 4). The intramolecular disulfide bonds are presumed to be important for protein folding, stability, and protection of such proteins in the harsh acidic and protease-rich apoplast, when they are delivered to plant cells during infection by pathogens [57]. Thus, the correct folding or stability of the disulfide bonds in VdSCP76 and VdSCP77 are important for their functioning during host-*V. dahliae* interactions.

In this study, the CFEM-containing proteins displayed differentiation in their virulence function on cotton. The majority of deletion mutants failed to affect virulence, though several increased susceptibilities to *B. cinerea* after transient expression of VdSCPs (Fig. 6). It is apparent that VdSCP76 and VdSCP77 contribute to virulence by suppressing immunity since overexpressing either *VdSCP76* or *VdSCP77* in *N. benthamiana* enhanced its susceptibility to *B. cinerea* (Fig. 3D–F). A similar role that contributes to virulence has been demonstrated for PpEC23 in *P. pachyrhizi* and Pst_12806 in *Puccinia striiformis* f. sp. *tritici* (*Pst*) [7, 9]. However, except for VdSCP99, gene deletion and transient expression of the other six CFEM family members failed to yield a role in virulence, but their virulence function may be concealed by the lower efficiency of immunity suppression or the redundant function of other CFEM family members when deleted. Together, our study showed that the CFEM family members in *V. dahliae* displayed differences in virulence function.

CFEM proteins have been intensively studied in *C. albicans* [58–61], demonstrating that CFEM proteins bind to the haem-Fe^3+^ and extract it from the host hemoglobin, as well as in mediating iron delivery into the *C. albicans* cell [31–35, 61, 62]. Iron acquisition is essential for the virulence of many plant pathogens, and therefore, pathogens employ diverse strategies to sequester iron from plants [37, 63]. However, the host may also use iron to increase local oxidative stress in defense responses against pathogens in a Fenton reaction to form deleterious reactive oxygen [37]. Iron-starved *Arabidopsis* plants displayed higher resistance to the bacterial pathogen *Dickeya dadantii* and the necrotrophic fungus *B. cinerea* [64], but the maize pathogen *C. graminicola* displays higher virulence on iron-starved plants than in iron-sufficient plants, as the iron-deprived plants stimulate a weaker oxidative burst at the site of pathogen infection [65]. In *V. dahliae*, the pathogen displays higher aggression on several iron-deficient hosts in contrast to iron-sufficient hosts [39, 40].

Sequestration of iron from host plants is likely to be important for host colonization by *V. dahliae* [66]. In this study, transiently expressed CFEM family members with a conserved iron binding site (Asp-type, with negative charged side chain) affect the expression of iron homeostasis-related genes in *N. benthamina*, but lost the ability to alter the expression of iron homeostasis-related genes coincident with the mutation in the conserved iron binding site to an Asn residue (Asn-type, VdSCP76 and VdSCP77) (Fig. 7E, F). However, the Asn residue in the iron-chelating site seems necessary but not sufficient for functional manipulation of immunity since the mutation (D>>N) cannot fully suppress immunity as the Asn-type (Additional file 2: Fig. S13). Moreover, the functional divergence of CFEM proteins that fall into the Asp-type and Asn-type are conserved in some pathogenic fungi, such as *Fusarium oxysporum* or *M. oryzae* (Additional file 2: Fig. S15). Therefore, *V. dahliae*, and some other pathogenetic fungi likely deploy some CFEM family members (Asp-type) to balance the iron sequestration and others (Asn-type) for suppression of host immunity to promote successful colonization and propagation by *V. dahliae* in the iron-deficient environment of host xylem (Fig. 8).

## Conclusions

In conclusion, the functions of nine CFEM-containing VdSCPs from the plant pathogenic fungus *V. dahliae* were identified, and these have differential roles in virulence. VdSCP76 and VdSCP77 showed the broadest-spectrum immunity suppression ability and highest virulence related to the potential conversed iron binding site mutation (Asp>>Asn), compared to other seven CFEM-containing VdSCPs that probably involves in iron predation. Thus, the CFEM family members of *V. dahliae* that likely diverged into Asn and Asp types with different functions in iron predation and immunity suppression, respectively. Both functions are necessary for the colonization and propagation of *V. dahliae* in the iron-insufficient environment of host plants. Future efforts will be devoted to investigating the mechanisms in which VdSCP76 and VdSCP77 suppress host immunity and whether the other seven CFEM members are involved in iron predation in the iron-deprived environment witin the plant vascular system.

## Methods

### Identification of CFEM-containing VdSCPs in Verticillium dahliae Vd991

Candidate CFEM-containing VdSCPs were predicted using an HMMER package with the HMM profile of the CFEM domain (Pfam ID: PF05730) queried against the *V. dahliae* Vd991 genome using the default parameters. The returned hits with *E*-values < 1e−10 and Scores > 30 were manually selected. Secreted proteins were identified using four programs commonly used to identify protein localization, as described previously [27]. Putative extracellular proteins containing a signal peptide but lacking transmembrane domains were identified as secreted proteins. The orthologous genes in the *V. dahliae* VdLs.17 genome were determined by BLAST [67]. Clustal X2 and Clustal W2 were used for multiple sequence alignment [48].

### Growth of microbes and plant material

The *V. dahliae* wild-type strain Vd991 (highly virulent isolate from *Gossypium hirsutum*) was cultured on potato dextrose agar (PDA) or in liquid Czapek-Dox medium for 5 days at 25 °C. The *V. dahliae* genetic transformants constructed in this study were cultured on PDA with 50 μg/mL hygromycin B. *Botrytis cinerea* strain B05.10 was grown on PDA medium at 25 °C. *Agrobacterium tumefaciens* AGL-1 and GV3101 were cultured in Luria-Bertani medium (LB, Tryptone 10 g, NaCl 10 g, Yeast extract 5 g, ddH_2_O 1000 mL) at 28 °C for fungal transformations and transient expression experiments in plants, respectively. Cotton (*Gossypium hirsutum* cv. Junmian No.1) seedlings were grown at 25 °C for 3 weeks for virulence assays. Tobacco (*Nicotiana benthamiana* LAB) seedlings were grown at 25 °C for 4 weeks for transient expression experiments. Both cotton and tobacco plants were grown in a greenhouse with 14-h light/10-h dark photoperiod in at the temperatures noted above.

### Transient expression in *Nicotiana benthamiana*

The CFEM-containing *VdSCPs* genes were amplified from *V. dahliae* Vd991 strain cDNA using the indicated primers (Additional file 1: Table S3). To study the functions of domains encoded within the CFEM-containing VdSCPs genes, the following were prepared: VdSCP76 without the CFEM domain (*VdSCP76-*Δ*C*); VdSCP76 and VdSCP77 which retained the signal peptide and CFEM domain sequences (*VdSCP76-C* and *VdSCP77-C*), and the genes without their signal peptides (*VdSCP76*^Δ*SP*^ and *VdSCP77*^Δ*SP*^). The site-directed mutagenesis of amino acid residues critical to the function of *VdSCP76* (*VdSCP76*^*N43D*^, *VdSCP76*^*CnA*^) and *VdSCP77* (*VdSCP77*^*N47D*^, *VdSCP77*^*CnA*^) were performed using a Fast Mutagenesis System Kit (TransGen, Beijing, China). All sequences were cloned severally into the PVX vector pGR107 with the ClonExpress II One-Step Cloning Kit (Vazyme, Nanjing, China) according to the manufacturer’s instructions, and then were transformed into the *A. tumefaciens* GV3101 and were grown in LB medium at 28 °C overnight, respectively. Cells were harvested and resuspended in salt solution with 10 mM MgCl_2_, 10 mM MES, and 200 μM acetosyringone, pH 5.6. To examine the suppression of cell death induction in *N. benthamiana*, two *A. tumefaciens* cells carrying appropriate constructs (targeted genes and cell death-inducing genes) were mixed at 1:1 ratio to OD600 = 0.8 for each and for co-transient expression in 4-week-old *N. benthamiana* leaves. The Bcl-2-associated X protein (BAX) and green fluorescent protein (GFP) as positive and negative controls, respectively. Symptom development was monitored at 3 days in a time-course experiment until 6 days post-infiltration (dpi). To verify protein production during transient expression in *N. benthamiana*, total proteins were extracted using the P-PER Plant Protein Extraction Kit (Thermo Scientific, USA) and Protease Inhibitor Cocktail Kit (Thermo Scientific, USA) from the agro-infiltrated *N. benthamiana* leaves 60 h after inoculation, following the manufacturer’s instructions. The proteins were separated using 12% sodium dodecyl sulfate polyacrylamide electrophoresis gels, and transient protein expression in *N. benthamiana* was verified using anti-FLAG antibody (Sigma-Aldrich, USA).

### Yeast signal sequence trap system

Functional validation of the predicted signal peptide was performed as described previously [43]. The predicted signal peptide encoding sequences of *VdSCP76* and *VdSCP77* were fused in frame to the vector pSUC2. The recombinant constructs, pSUC2::SP^VdSCP76^ and pSUC2::SP^VdSCP77^, were transformed into the yeast strain YTK12 respectively and screened on CMD-W (lacking tryptophan) medium. Positive clones were confirmed by PCR using vector-specific primers (Additional file 1: Table S3). The positive transformants were incubated on YPRAA medium containing 2% raffinose. The recombinant YTK12 strain carrying the signal peptide sequence of *Avr1b* (pSUC2::SP^Avr1b^) was used as positive control, while the untransformed YTK12 strain and YTK12 strain with an empty pSUC2 vector were used as negative controls.

### Subcellular localization assays

To study the subcellular localization of VdSCP76 or VdSCP77 *in planta*, the wild-type gene *VdSCP76* and *VdSCP77* fused mCherry sequence were introduced into the vector pCAMBIA1300-35S. *Agrobacterium tumefaciens* Gv3101 strain carrying pCAMBIA1300-35S-VdSCP76-mCherry or pCAMBIA1300-35S-VdSCP77-mCherry was infiltrated into 3-week-old *N. benthamiana* leaves. The *A. tumefaciens* Gv3101 carrying pCAMBIA1300-35S-mCherry was used as control. To further examine the subcellular localization of VdSCP76 or VdSCP77, a fused fragment comprising *TrpC*-promoter region, coding sequence of *VdSCP76* or *VdSCP77*, *GFP* sequence, and *Nos*-terminator was introduced into the donor vector pCOM that carries geneticin resistance [68]. Positive recombinant vectors were transferred into *A. tumefaciens* strain AGL-1 for fungal transformation and complemented transformants were selected and isolated on potato dextrose agar (PDA) medium with geneticin antibiotics. The positive transformants were verified by a PCR method with the appropriate test primer pairs (Additional file 1: Table S3). Onion epidermal cells infected with *V. dahliae* expressing VdSCP76-GFP or VdSCP77-GFP fusion proteins were examined by confocal microscopy.

To observe fluorescence, the infiltrated *N. benthamiana* leaves were harvested 2 days post-agro-infiltration and onion epidermal cells were harvested at 4 days after incubation, and directly imaged under a Leica TCS SP8 confocal microscope with an excitation wavelength of 580 nm, emission of 610 nm for mCherry and excitation wavelength of 488 nm, emission of 510 nm for GFP, excitation of 543 nm and emission at 562 nm for FM4-64 dye, respectively.

### ROS and callose staining, and electrolyte leakage detection

The elicitor activity was detected by single infiltration and co-infiltration of VdSCP76 or VdSCP77 with VdEG1 in *N. benthamiana* leaves; VdEG1 and GFP were used as positive and negative controls respectively. The following indexes were detected at 60 h after infiltration in *N. benthamiana* leaves. Reactive oxygen species (ROS) generation in *N. benthamiana* leaves was detected using 3’3-diaminobenzidine (DAB) solution as described previously [69]. Callose deposition was determined under fluorescence microscopy using a UV filter and the numbers of light spots were counted using ImageJ software after decoloring with 95% ethanol and then incubating in 150 mM phosphate buffer (pH 9.5) containing aniline blue (approximately 1% w/v; Sigma) for 2 h in the dark [70]. Electrolyte leakage assays were performed as described previously [71], and ion conductivity was then measured using a conductivity meter with Probe LE703 (Mettler-Toledo, Shanghai, China).

### Gene expression analysis

In this experiment, the EASYspin Plus RNA Rapid Extraction Kit (Aidlab Biotech, Beijing, China) including genomic DNA (gDNA) removal procedures were used to isolate total RNA, and the RNA was examined for lack of gDNA contamination using primer pairs for the *V. dahliae VdEF-1α*. The cDNA was synthesized using cDNA synthesis supermix (TransGen Biotech, Beijing, China) following the manufacturer’s instructions. The specificity of primers was assessed by melt curve analysis (single peaks) of amplicons. The primer pair amplification efficiencies of each gene were assessed using standard procedures.

To determine the expression of nine CFEM-containing VdSCPs during infection, 3-week-old cotton seedlings were root-dip-inoculated with 1 × 10^7^ conidia/mL *V. dahliae* and the roots were harvested at different time points. Cycling parameters for SYBR Green-based reverse transcription-quantitative PCR (RT-qPCR) included the following: an initial 95 °C denaturation step for 5 min, followed by denaturation for 15 s at 95 °C, annealing for 40 s at 60 °C, and extension for 30 s at 72 °C for 40 cycles. The *V. dahliae* elongation factor 1-α (*EF-1α*) (Additional file 1: Table S3) was used as endogenous reference.

The transient expression samples were also collected at 2 days after single- or co-infiltration of GFP, VdEG1, VdSCP76, and VdSCP77 in *N. benthamiana* leaves, for the detection of resistance-related genes. The *N. benthamiana* elongation factor 1-α (*NbEF-1α*) (Additional file 1: Table S3) was used as endogenous reference, and the primer pairs of detected resistance-related genes are listed in Additional file 1: Table S3. The RT-qPCR was performed with an initial 95 °C denaturation step for 5 min, followed by denaturation for 30 s at 95 °C, annealing for 30 s at 60 °C, and extension for 30 s at 72 °C for 40 cycles.

To detect gene expression of CFEM-containing SCPs under iron starvation (150 μM Bathophenanthrolinedisulfonic acid disodium salt, BPS) and under sufficient iron (50 μM FeCl_3_) conditions, *V. dahliae* strains were cultured on Czapek-Dox medium with exogenous additives above and the mycelium was collected after 3 days of induction. The *V. dahliae EF-1α* was used as endogenous reference. All primer pairs used are listed in Additional file 1: Table S3. The relative expression of SCP CFEM-containing gene family members in *V. dahliae* was examined by RT-qPCR with an initial 95 °C denaturation step for 5 min, followed by denaturation for 15 s at 95°C, annealing for 40 s at 60 °C and extension for 30 s at 72 °C for 40 cycles.

All qPCR or RT-qPCR experiments were repeated two or three biological replicates, and each biological replicate experiment contained three technical replicates. Relative transcript levels of different genes among various samples were evaluated using the 2^−∆∆CT^ method [72].

### Fungal transformations for gene deletion and complementation

For targeted gene deletions in *V. dahliae*, the gene deletion construct was generated comprising the approximately 1 kb 5′ and 3′ flanking regions of targeted gene sequence in pGKO2-*Hyg* vector. To generate complementation transformants, the sequences including the native promoter region, gene sequence (the wild-type *VdSCP76* or *VdSCP77*, site-directed mutagenized genes *VdSCP76*^*N43D*^ or *VdSCP77*^*N47D*^), and native terminator region of targeted gene were amplified and introduced into the binary vector pCOM that carries geneticin resistance [68]. For the subcellular localization of VdSCP76 or VdSCP77 in natural secretion, a fused fragment including *TrpC*-promoter region, coding sequence of *VdSCP76*-*GFP* or *VdSCP77*-*GFP*, and the *Nos*-terminator was introduced into the vector pCOM [68]. The corresponding amplification primer pairs of targeted gene deletion complementation are listed in Additional file 1: Table S3.

The positive recombinant vectors were transferred into *A. tumefaciens* strain AGL-1 for the fungal transformation. Gene deletion and complementation transformants were generated by *A. tumefaciens*-mediated transformation (ATMT) method described previously [73]. The positive transformants were screened and isolated on potato dextrose agar (PDA, 200 g/L potato, 20 g/L glucose, 15 g/L agar) medium containing 50 μg/mL hygromycin for gene deletion and 50 μg/mL geneticin for complementation and were verified by PCR with the appropriate test primer pairs (Additional file 1: Table S3).

### Virulence assays

Four-week-old *N. benthamiana* leaves were infiltrated with *A. tumefaciens* Gv3101 carrying the vector harboring GFP as control on the left of leaves and carrying the recombinant vector harboring CFEM-containing VdSCPs on the right of leaves. The center of the infiltration area of leaves were inoculated with 10 μL 5 × 10^6^ conidia/mL *Botrytis cinerea* conidial suspension after 12 h of agro-infiltration. The inoculated plants were placed in an incubator at 25 °C and at 80% relative humidity. The lesion diameters were measured at 4 days. SYBR Green-based qPCR was used to detect the biomass of *Botrytis cinerea* with an initial 95 °C denaturation step for 5 min, followed by denaturation for 15 s at 95 °C, annealing for 40 s at 60°C, and extension for 30 s at 72 °C for 40 cycles. The *B. cinerea* actin gene was used to quantify fungal colonization and the *N. benthamiana EF-1α* gene served as an endogenous control. The primers are listed in Additional file 1: Table S3.

Virulence assays were performed on cotton seedlings as previously described [73]. Three-week-old cotton seedlings were inoculated with 5 × 10^6^ conidia/mL by a root-dip method [73]. The disease index (DI) was calculated as = [Σ (the seedling of every grade × relative grade)/(total seedlings × the most serious grade)] × 100 following Powell et al. [74]. Vascular discoloration of infected cotton was observed in longitudinal sections of the shoots 3 weeks after inoculation. Fungal biomass in cotton was determined by SYBR Green-based qPCR as described previously [75] with the primer pairs listed in Additional file 1: Table S3. The *V. dahliae EF-1α* gene was used to quantify fungal colonization, and the cotton *18S* gene served as an endogenous control. At least 20 cotton seedlings were inoculated for each treatment and the experiment was conducted twice. One-way analysis of variance (ANOVA) was performed to determine statistical significance at *P* < 0.01 between treatments and the wild-type.

## Supplementary Information

**Additional file 1: Table S1.** Bioinformatic predictions on the secretory characteristics of nine VdSCPs in *V. dahliae* related with suppression activity for VdEG1. **Table S2.** Bioinformatic predictions on the secretory characteristics of CFEM – containing VdSCPs in *V. dahliae*. **Table S3.** Oligonucleotide primers used in this study.

**Additional file 2: Figure S1.** Analysis of the cell death suppression activities of 120 VdSCPs in *Nicotiana benthamiana* leaves. **Figure S2.** Analysis of the broad-spectrum cell death suppression activities of CFEM-containing VdSCPs in *Nicotiana benthamiana* leaves. **Figure S3.** Analysis of signal peptide function and subcellular localization of VdSCP76 and VdSCP77. **Figure S4.** Polymerase chain reaction (PCR) analysis of gene deletion transformants of CFEM-containing VdSCPs from *V. dahliae*. **Figure S5.** Polymerase chain reaction (PCR) analysis of gene complementation transformants. **Figure S6.** Identification of the immunity suppression activity of VdSCP76 and VdSCP77 in *Nicotiana benthamiana*. **Figure S7.** Immunoblotting analysis of proteins in *Nicotiana benthamiana* leaves transiently expressing VdEG1 in the suppression experiment. **Figure S8.** Pathogenicity assay of double gene deletion of VdSCP76 and VdSCP77 strains on cotton. **Figure S9.** Functional diversification analysis of CFEM-containing VdSCPs family members. **Figure S10.** Gene expression of CFEM-containing VdSCPs family members during infection of cotton roots. **Figure S11.** Analysis of defense related-gene expression level. **Figure S12.** The fungal biomass and disease index of indicated strains on cotton. **Figure S13.** Functional analyses of conserved asparagine residues in the CFEM domain-containing members from *Verticillium dahliae*. **Figure S14.** Gene expression level of CFEM-containing VdSCPs under iron starvation and ferric ion conditions. **Figure S15.** Functional dissection Asp-type and Asn-type CFEM-containing secretory proteins from *Fusarium oxysporum* and *Magnaporthe oryzae*.

**Additional file 3:** Individual data points.

## Data Availability

All study data are included in the article and/or supplementary information.
