# Supplementary material for: Verticillium dahliae CFEM proteins manipulate host immunity and differentially contribute to virulence
Source: BMC Biol. 2022 Feb 23;20:55. doi: 10.1186/s12915-022-01254-x (PMC8867779; doi:10.1186/s12915-022-01254-x)
Supplement: Supplementary file 2 — Additional file 2: Figure S1. Analysis of the cell death suppression activities of 120 VdSCPs in Nicotiana benthamiana leaves. Figure S2. Analysis of the broad-spectrum cell death suppression activities of CFEM-containing VdSCPs in Nicotiana benthamiana leaves. Figure S3. Analysis of signal peptide function and subcellular localization of VdSCP76 and VdSCP77. Figure S4. Polymerase chain reaction (PCR) analysis of gene deletion transformants of CFEM-containing VdSCPs from V. dahliae. Figure S5. Polymerase chain reaction (PCR) analysis of gene complementation transformants. Figure S6. Identification of the immunity suppression activity of VdSCP76 and VdSCP77 in Nicotiana benthamiana. Figure S7. Immunoblotting analysis of proteins in Nicotiana benthamiana leaves transiently expressing VdEG1 in the suppression experiment. Figure S8. Pathogenicity assay of double gene deletion of VdSCP76 and VdSCP77 strains on cotton. Figure S9. Functional diversification analysis of CFEM-containing VdSCPs family members. Figure S10. Gene expression of CFEM-containing VdSCPs family members during infection of cotton roots. Figure S11. Analysis of defense related-gene expression level. Figure S12. The fungal biomass and disease index of indicated strains on cotton. Figure S13. Functional analyses of conserved asparagine residues in the CFEM domain-containing members from Verticillium dahliae. Figure S14. Gene expression level of CFEM-containing VdSCPs under iron starvation and ferric ion conditions. Figure S15. Functional dissection Asp-type and Asn-type CFEM-containing secretory proteins from Fusarium oxysporum and Magnaporthe oryzae. [file 12915_2022_1254_MOESM2_ESM.docx]

**Supplementary Figures**

**Figure S1**


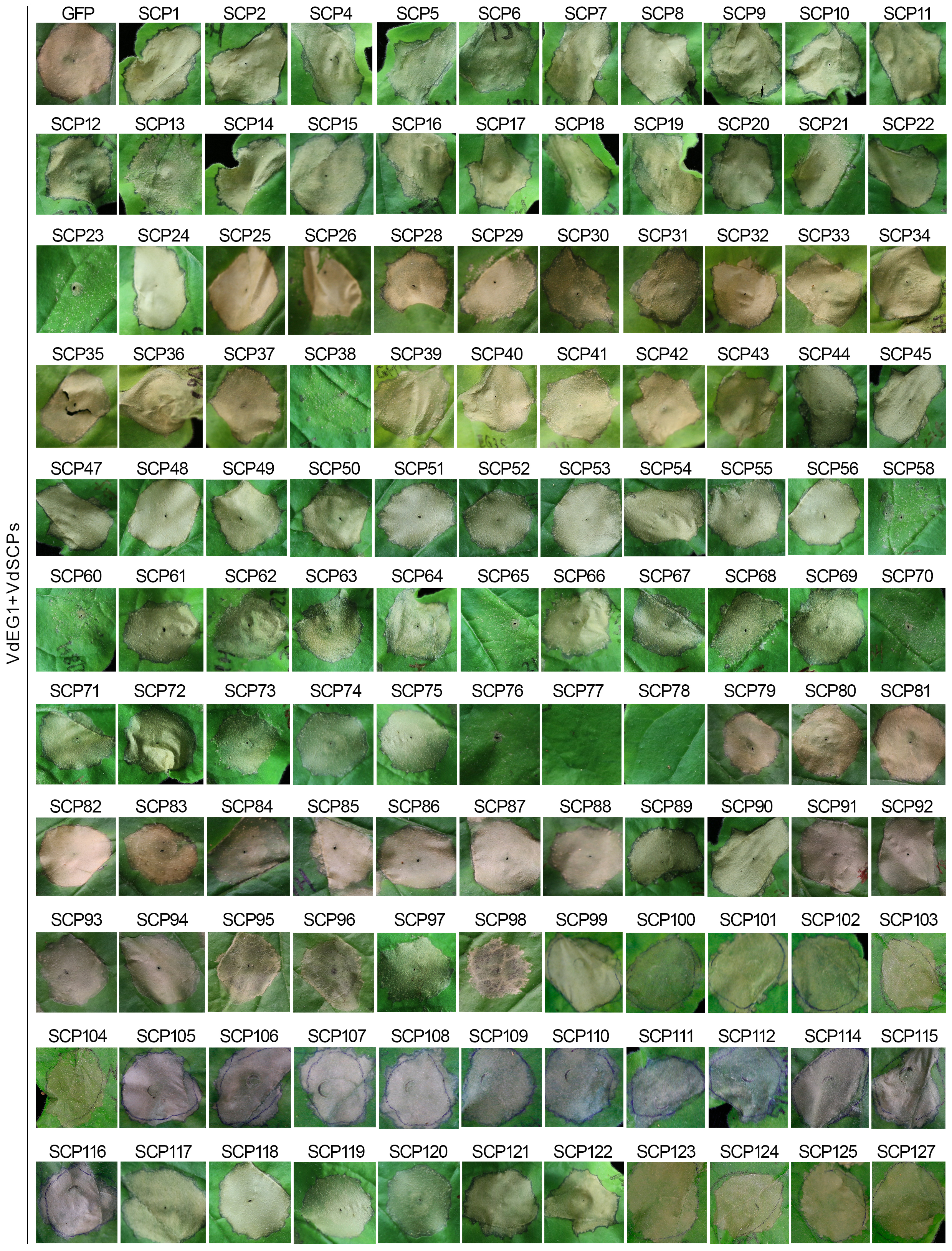


**Figure S1.** **Analysis of the cell death suppression activities of 120 VdSCPs in *Nicotiana benthamiana* leaves.** The suppression activity of 120 VdSCPs that there was no cell death inducing activity was detected by co-infiltration with the PAMP VdEG1 from *V. dahliae*. The detection was performed on the 4-week-old *N. benthamiana* leaves and the symptom development was monitored at 3 days in a time-course experiment until 6 dpi. The co-expression VdEG1 and GFP was used as the control.

**Figure S2**


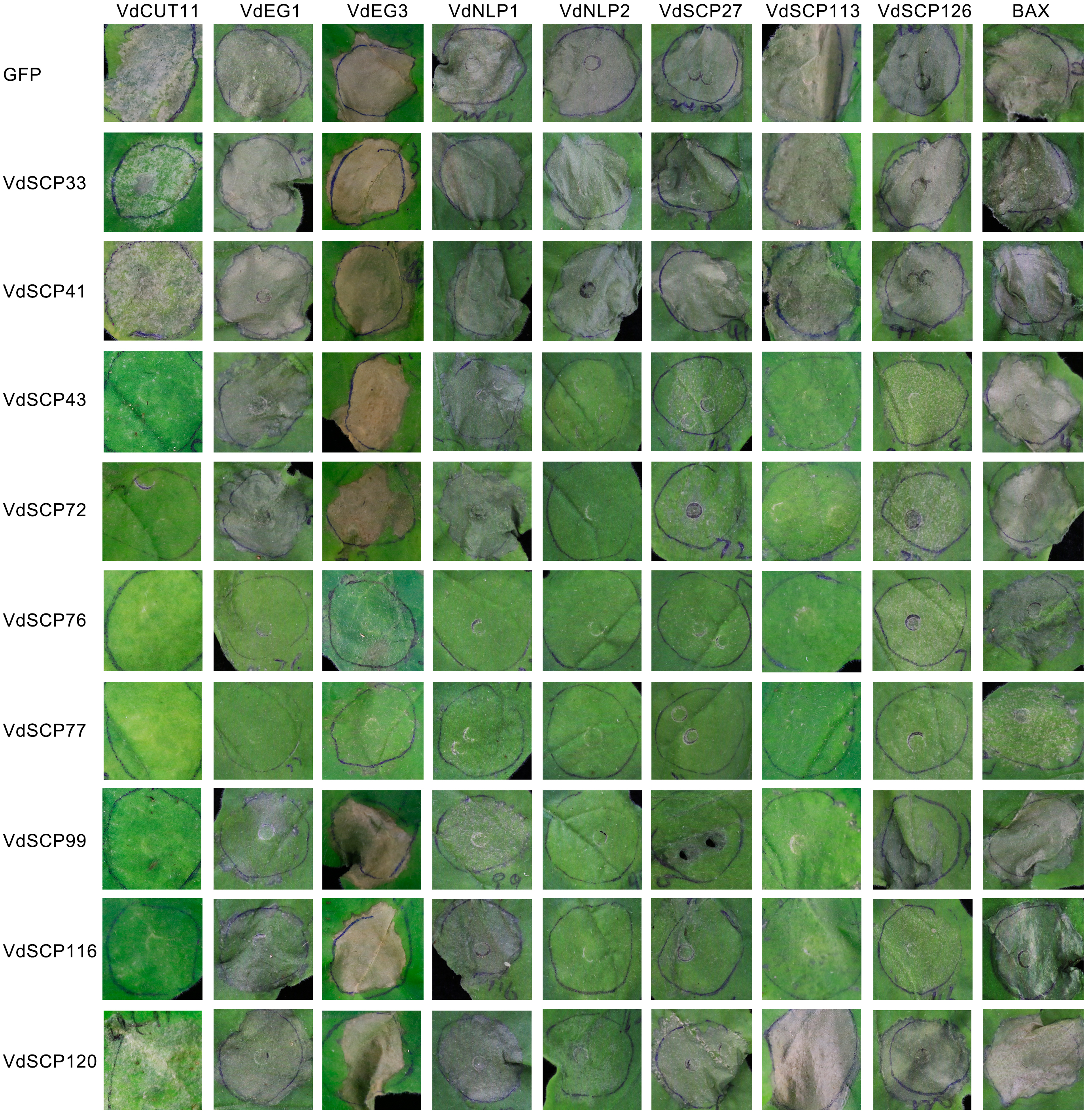


**Figure S2. Analysis of the broad-spectrum cell death suppression activities of CFEM-containing VdSCPs in *Nicotiana benthamiana* leaves.** The suppression activities of CFEM-containing VdSCPs against other identified *V. dahliae* cell death-inducing proteins VdSCP27, VdSCP113, VdSCP126, VdNLP1, VdNLP2, VdEG1 and VdEG3 were conducted in *N. benthamiana* leaves. The co-expression of CFEM-containing VdSCPs with Bcl-2-associated X protein (BAX) was used as control. The observation time was shown in Figure S1.

**Figure S3**


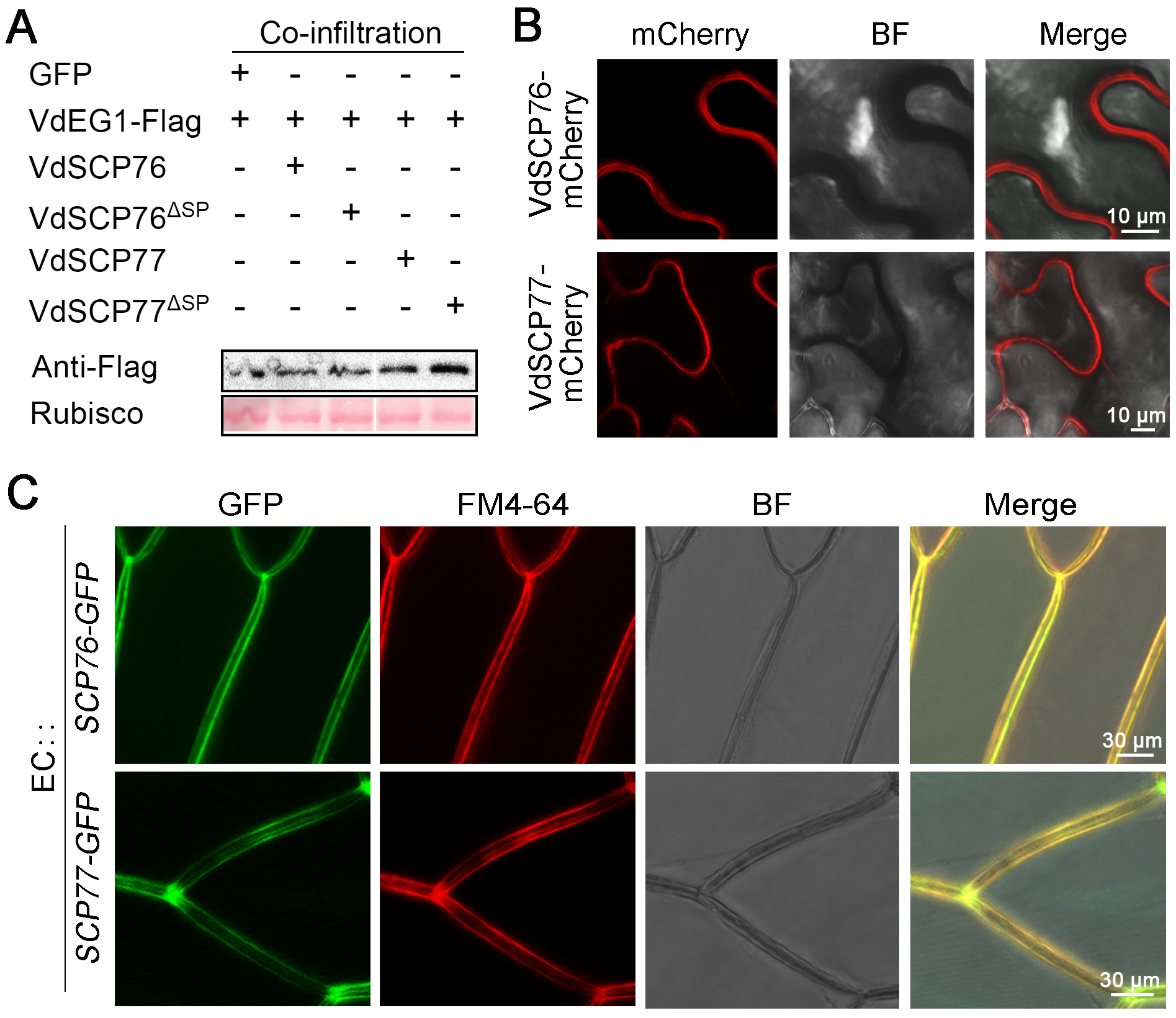


**Figure S3.** **Analysis of signal peptide function and subcellular localization of VdSCP76 and VdSCP77.** (**A**) Transient expression of VdEG1 in different co-expression treatments was validated by immunoblotting analysis. Ponceau S-stained Rubisco protein is shown as a total protein loading control. (**B**) Subcellular location of VdSCP76 or VdSCP77 fused with mCherry was analyzed by being expressed transiently in *N. benthamiana* leaves and the fluorescence was scanned by a Leica TCS SP8 confocal microscopy system with an excitation wavelength of 580 nm and emission of 610 nm. Bars = 10 μm. (**C**) The secretion and localization of VdSCP76 or VdSCP77 were analysed by co-incubation of conidial suspension of EC::*VdSCP76*-*GFP* or EC::*VdSCP77*-GFP with onion epidermal cells for 4 days. Wild type Vd991-GFP was used as control. The fluorescence was scanned by a Leica TCS SP8 confocal microscopy system with an excitation wavelength of 488 nm and emission of 510 nm for GFP, and excitation of 543 nm and emission at 562 nm for FM4-64 dye, respectively. Bars = 30 μm.

**Figure S4**


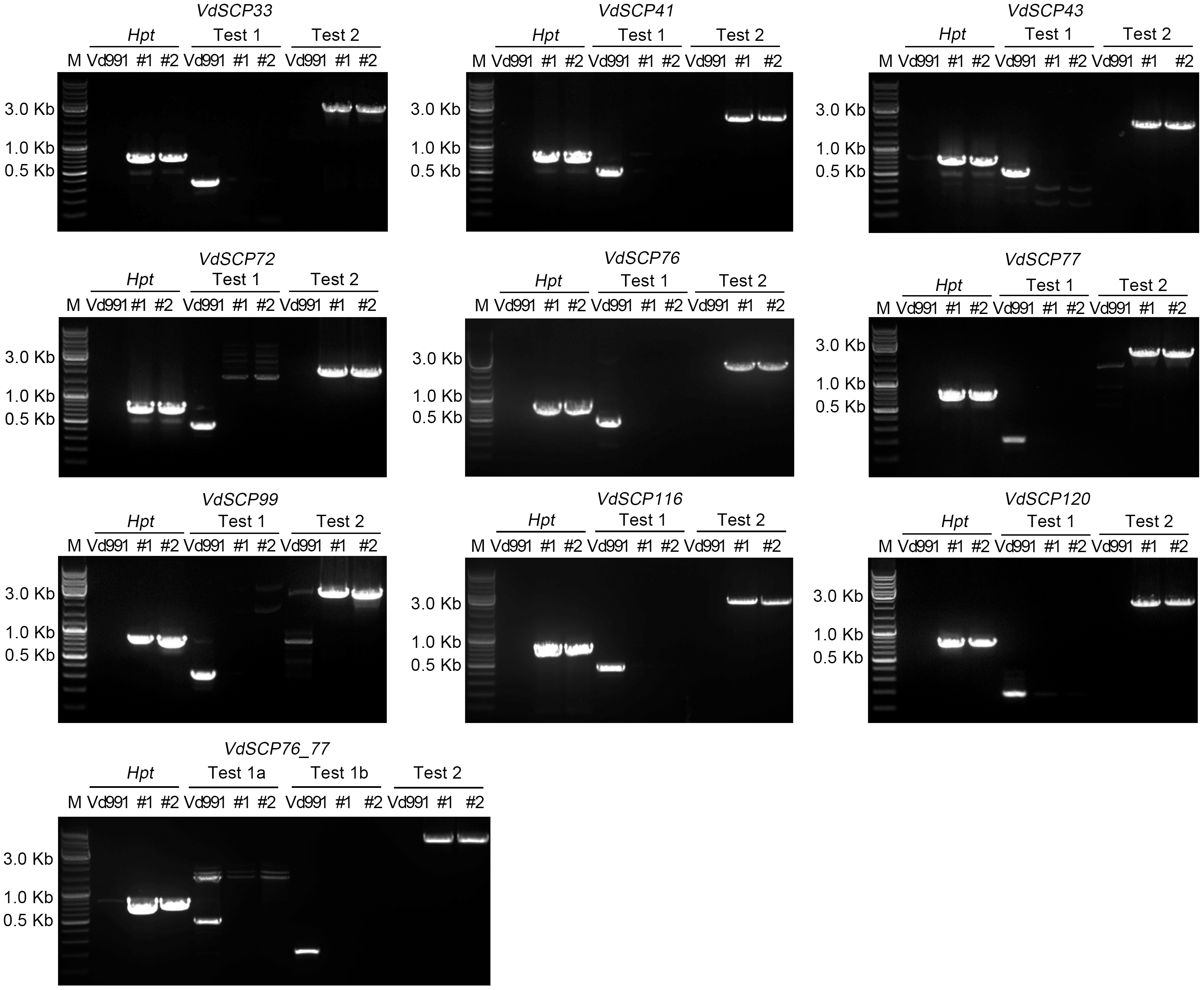


**Figure S4.** **Polymerase chain reaction (PCR) analysis of gene deletion transformants of CFEM-containing VdSCPs from *V. dahliae*.** Two transformants (#1 and #2) of each deletion mutant strain of CFEM-containing VdSCPs family members are shown. *Hpt*: PCR amplification of the positive selection marker hygromycin phosphotransferase gene. Test 1: PCR amplification of markers specific to the internal gene sequence. Test 2: PCR amplification of combination for the outer sequence upstream of target gene and the inner sequence of hygromycin phosphotransferase gene. WT: wild-type strain Vd991 was used as the control for hygromycin phosphotransferase gene and internal gene sequence marker, respectively. M=10,000 bp DNA ladder used as a size marker.

**Figure S5**


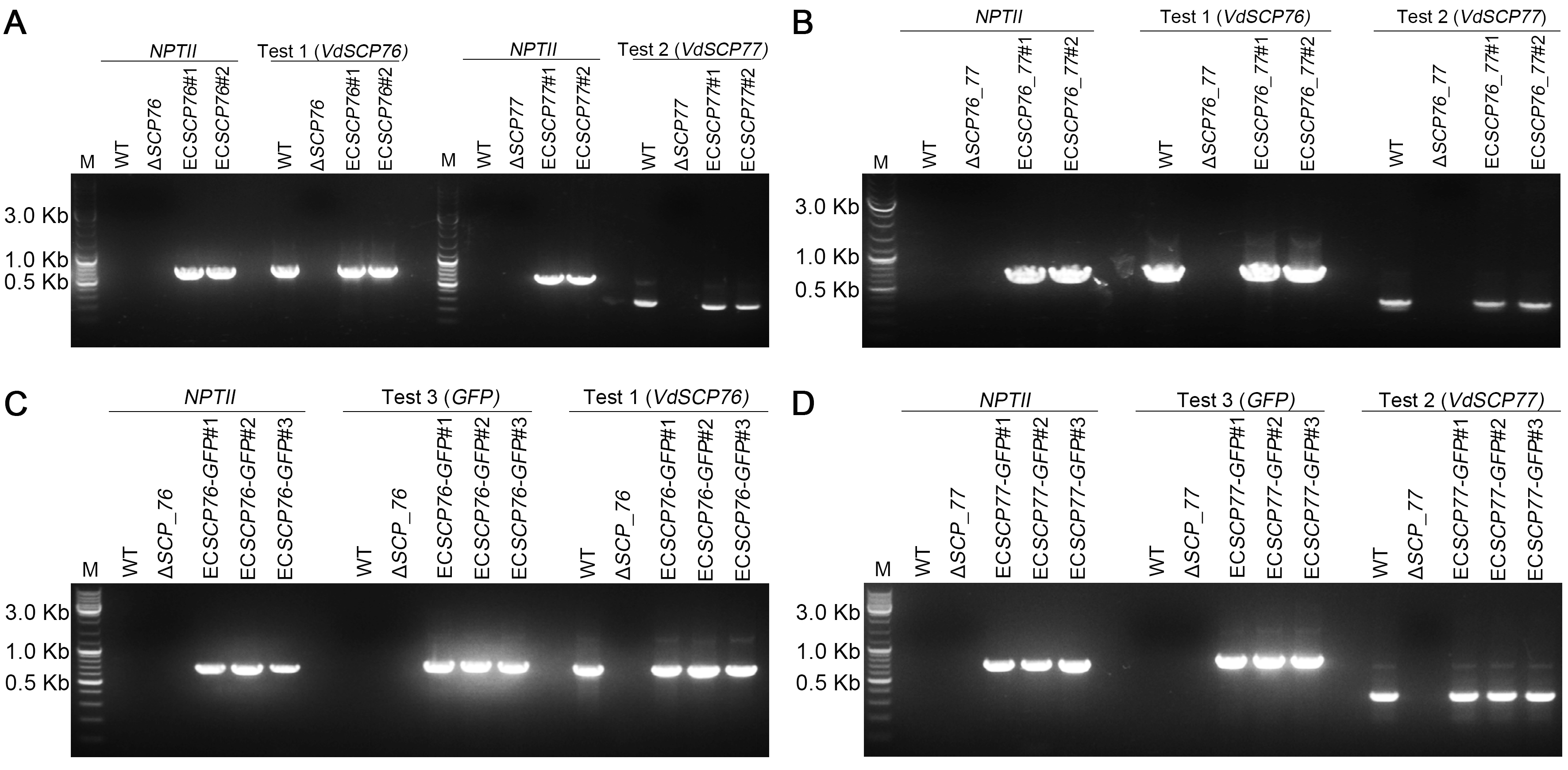


**Figure S5. Polymerase chain reaction (PCR) analysis of gene complementation transformants. (A)-(D)** #1, #2 and #3 represent independent complementation transformants of each strain. *NptII*: PCR amplification of the positive selection marker geneticin phosphotransferase gene. Test 1 and Test 2: PCR amplification of the internal gene sequence of *VdSCP76* and *VdSCP77* specially. Test 3: PCR amplification of the internal gene sequence of *GFP*. WT represents wild-type Vd991. M=10,000 bp DNA ladder used as a size marker.

**Figure S6**


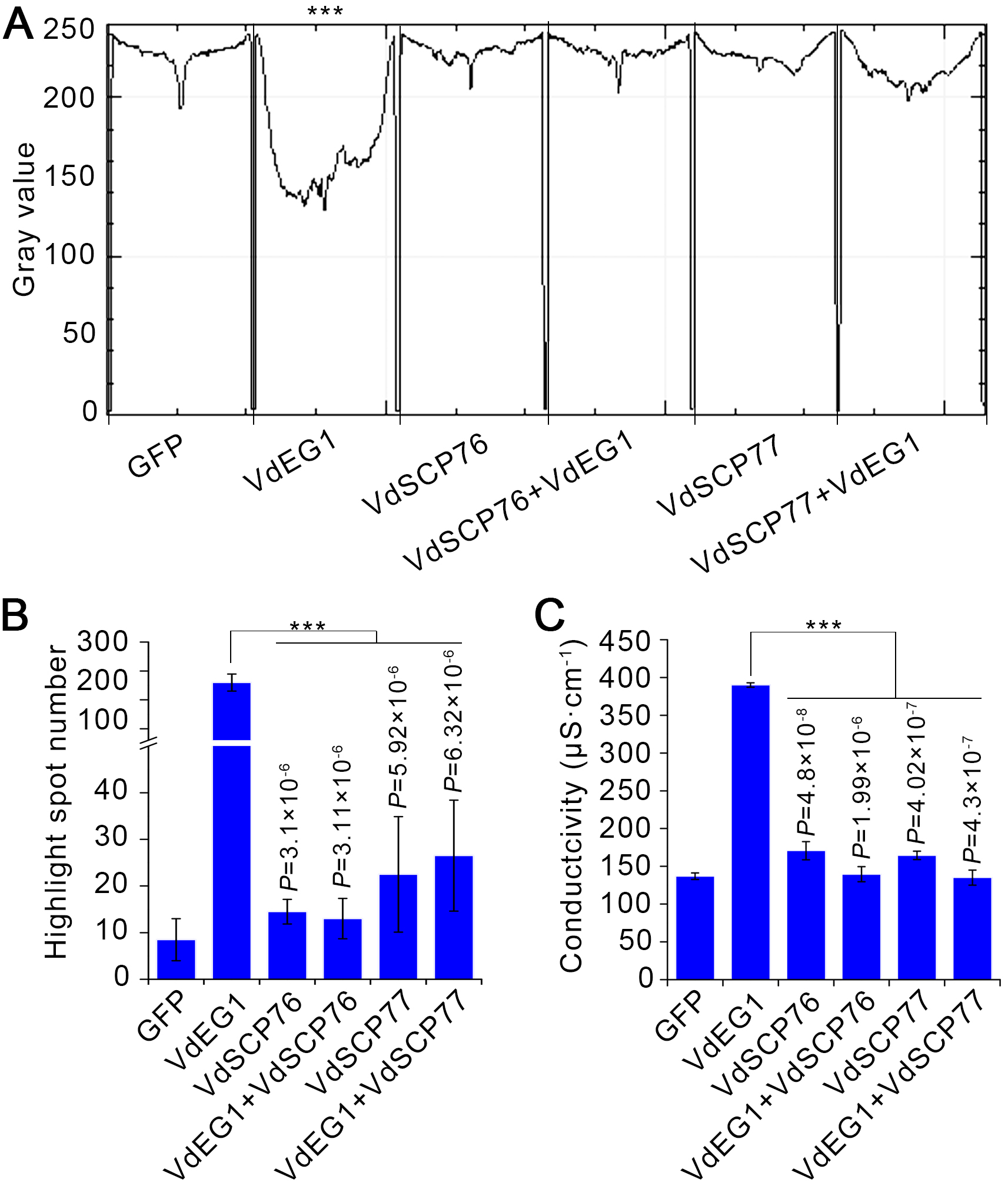


**Figure S6. Identification of the immunity suppression activity of VdSCP76 and VdSCP77 in *Nicotiana benthamiana*.** (**A**) ROS accumulation was detected using 3’3-diaminobenzidine (DAB) solution stained and the gray value was determined to indicate the level of ROS accumulation by ImageJ. (**B**) Callose deposition was determined using a fluorescence microscope using a UV filter after stained with aniline blue, and the spotlights were counted by ImageJ. (**C**) Electrolyte leakage was assessed by conductivity meter. All above treatments were performed in *N. benthamiana* leaves from 4-week-old plants 48 h after co-infiltration of VdEG1 with VdSCP76 or VdSCP77. VdEG1 and GFP were used as the controls. Error bars represent standard errors. *** represent statistical significance at *P* < 0.001 according to one-way analysis of variance (ANOVA) between VdEG1 and other treatments.

**Figure S7**


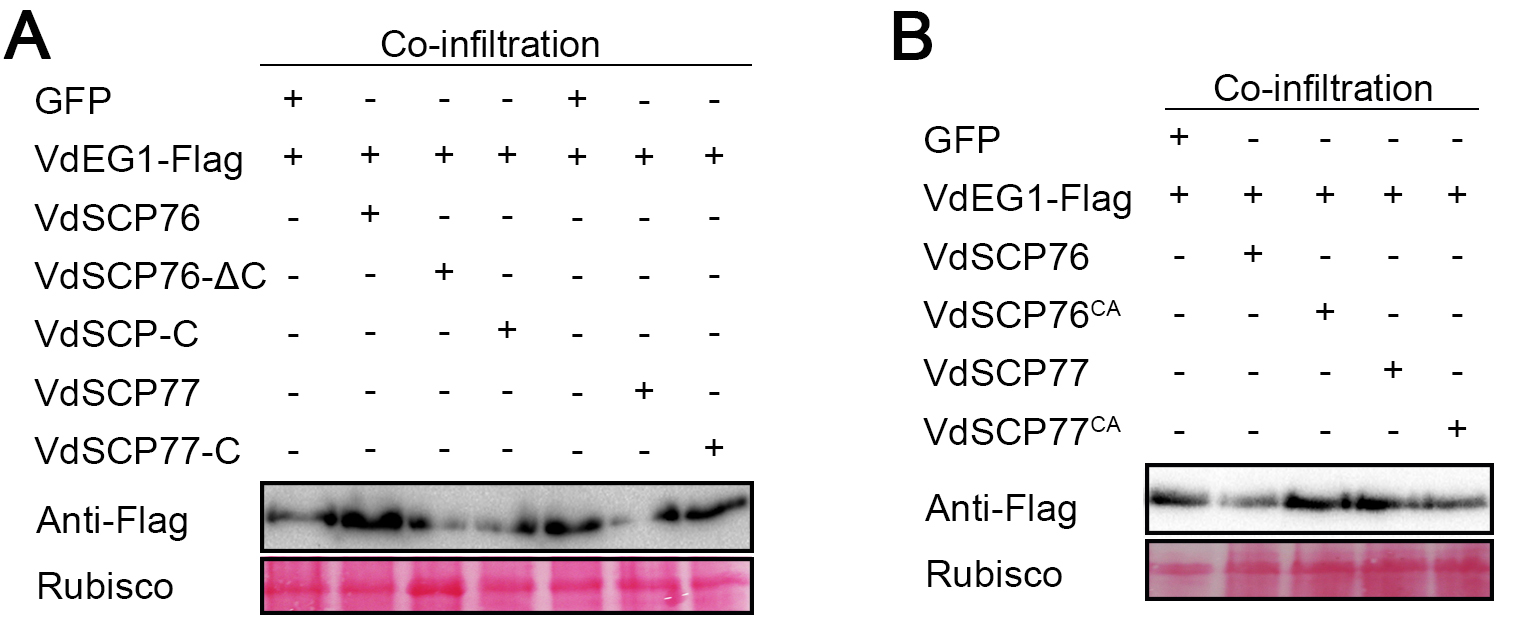


**Figure S7 Immunoblotting analysis of proteins in *Nicotiana benthamiana* leaves transiently expressing VdEG1 in the suppression experiment.** (**A**) and (**B**) The suppression experiments included transiently co-expression of truncated proteins or cysteine residue site mutated protein of VdSCP76 and VdSCP77 with VdEG1. Ponceau S-stained Rubisco protein is shown as a total protein loading control.

**Figure S8**


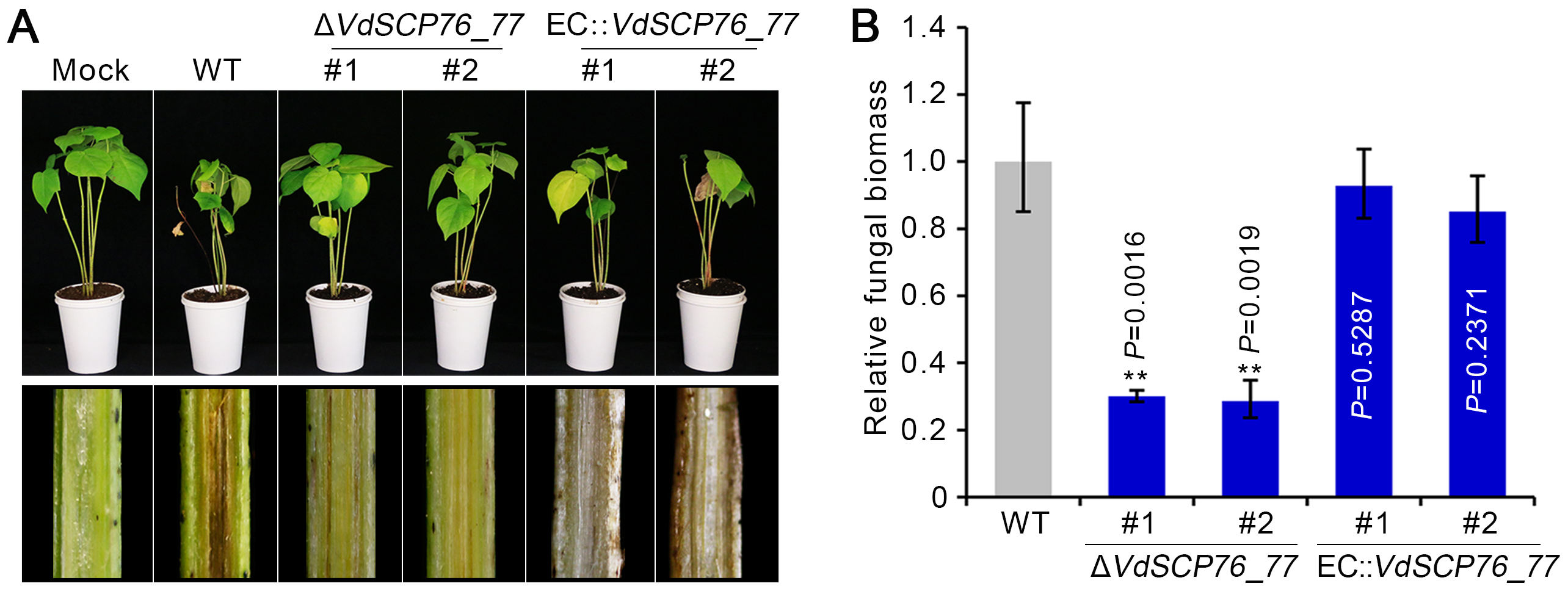


**Figure S8. Virulence assay of double gene deletion of *VdSCP76* and *VdSCP77* strains on cotton.** (**A**) Phenotypes of cotton seedlings inoculated with *VdSCP76* and *VdSCP77* double genes deletion mutants and complementary transformants were showed on the top and the stem longitudinal sections of cotton plants were showed on the bottom which were collected at 3 weeks post inoculation (wpi). (**B**) Fungal biomass of double genes deletion and complementary transformants on cotton seedlings was determined by qPCR. Error bars represent standard error. ** statistical significance at *P* < 0.01 in wild-type and each knockout mutant or complementary transformant according to one-way analysis of variance (ANOVA).

**Figure S9**


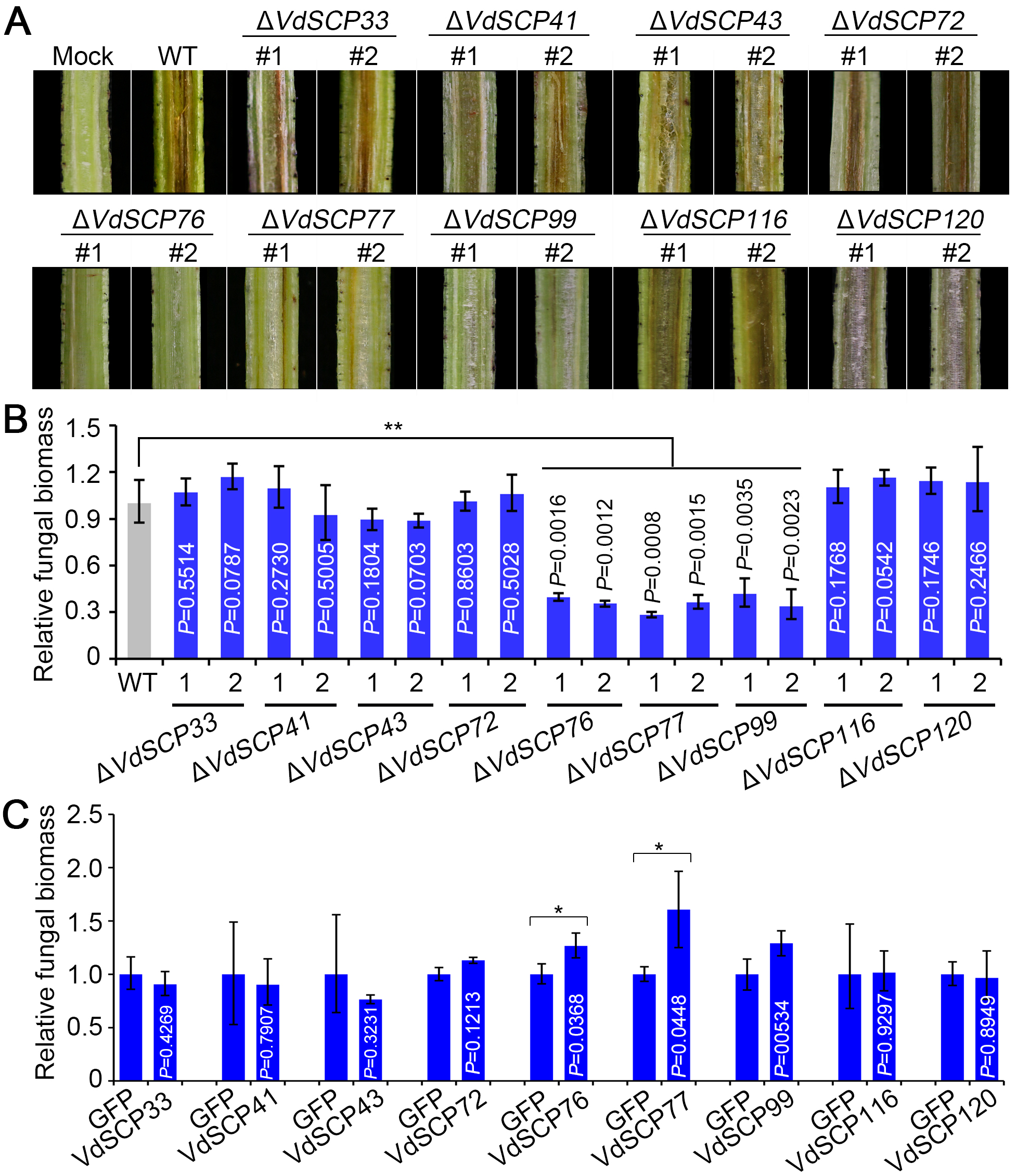


**Figure S9. Functional diversification analysis of CFEM-containing VdSCPs.** (**A**) Phenotypes of stem longitudinal sections of cotton seedlings inoculated with gene deletion mutants of CFEM-containing VdSCPs family members at 3 wpi. (**B**) Fungal biomass of gene deletion mutants of CFEM-containing VdSCPs family members was examined by qPCR. (**C**) Fungal biomass of *Botrytis cinerea* on *N. benthamiana* was evaluated by qPCR. Error bars represent standard error. * and ** represent statistical significance at *P* < 0.05 and *P* < 0.01, respectively, in control and different treatments (between wild-type and each knockout mutant, or GFP and CFEM family members) according to one-way analysis of variance (ANOVA).

**Figure S10**


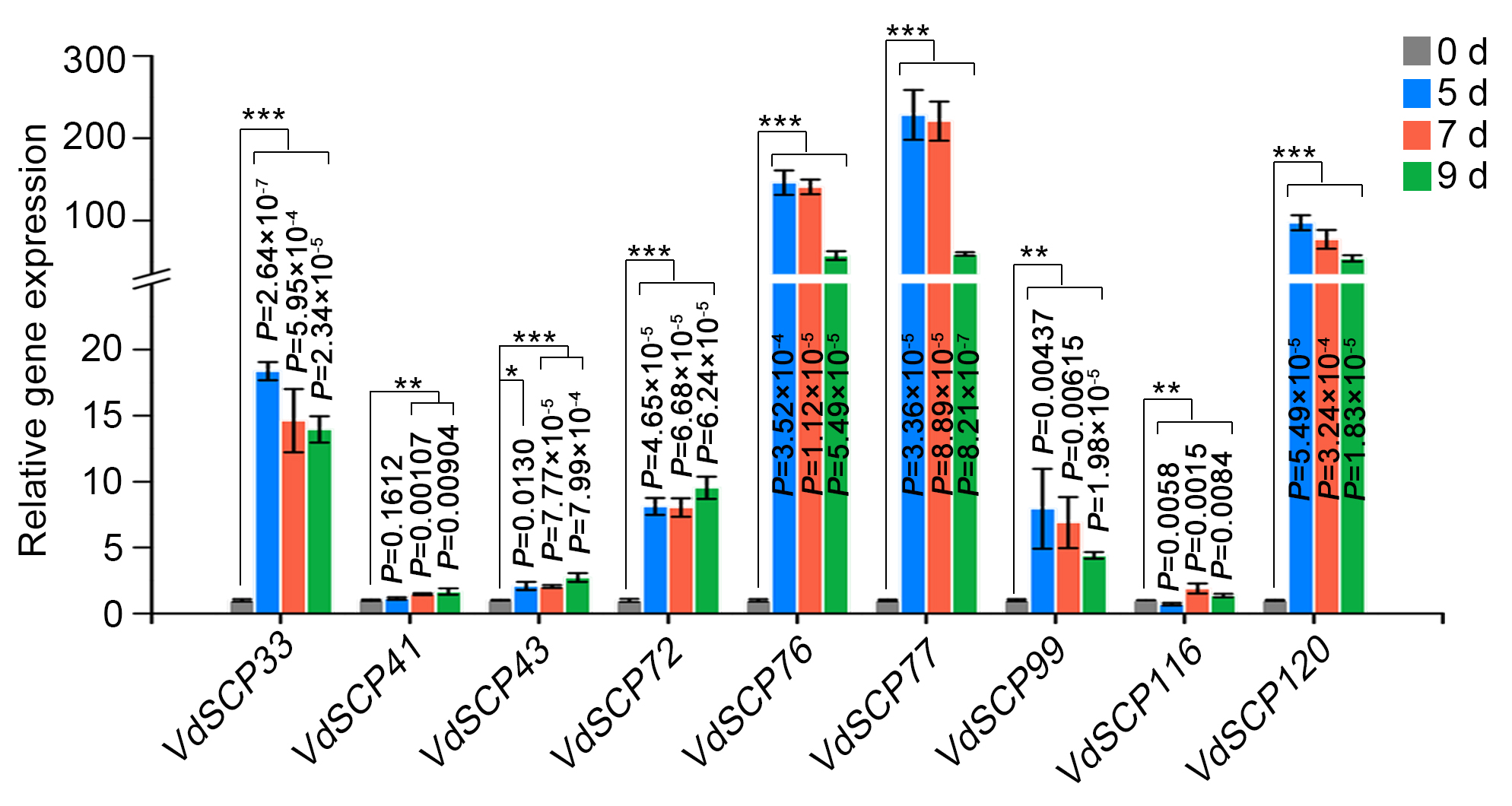


**Figure S10.** **Gene expression of CFEM-containing VdSCPs during infection of cotton roots.** Three-week-old cotton plants (cv. Junmian 1) were inoculated with wild-type *V. dahliae* Vd991 and harvested at 5, 7, 9 days after inoculation (dpi), respectively. RT-qPCR was performed to determine the gene expression levels of CFEM-containing VdSCPs family members relative to *V. dahliae* *EF-1α*. Error bars represent standard errors. *, ** and *** represent statistical significance at *P* < 0.05, *P* < 0.01 and *P* < 0.001, respectively, between 0 dpi and each time point of infection according to one-way analysis of variance (ANOVA) .

**Figure S11**


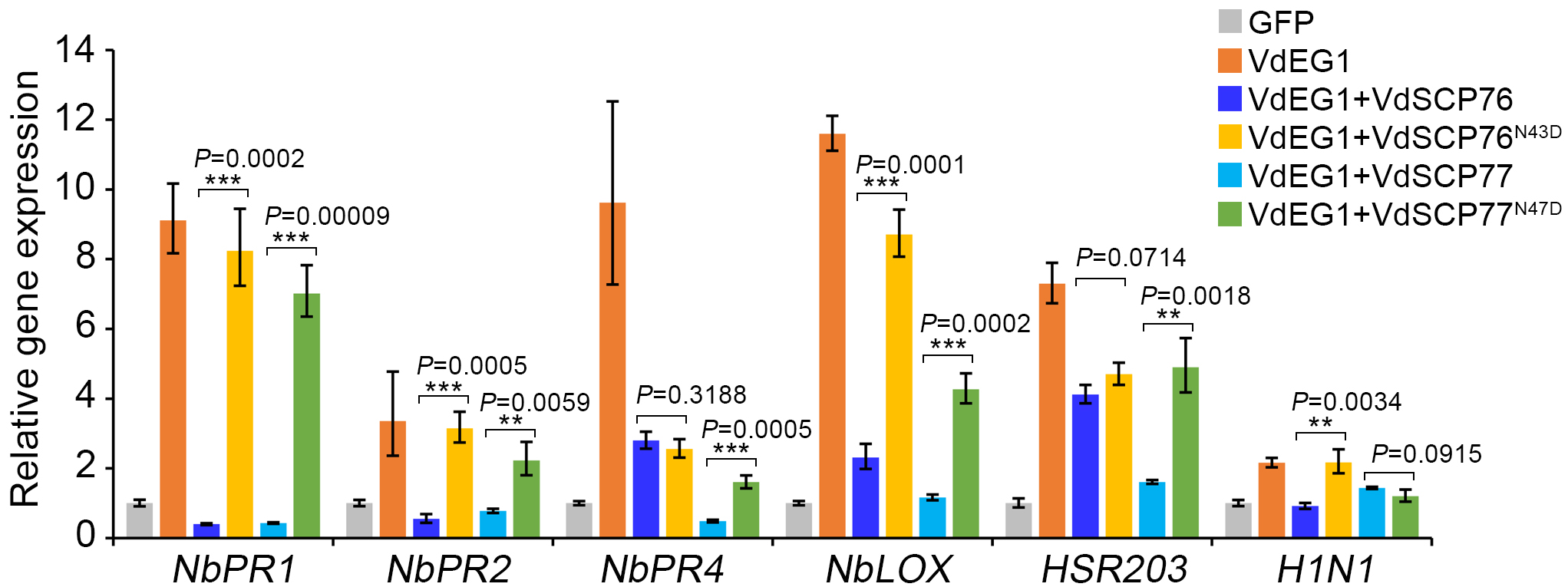


**Figure S11. Analysis of defense related-gene expression level.** Co-expression of VdSCP76, VdSCP77 and corresponding site-mutated proteins with VdEG1 transiently in *N. benthamiana* leaves, respectively, were conducted in 4-week-old *N. benthamiana* leaves. The transcripts were detected by RT-qPCR at 2 days after agro-infiltration. Single infiltration of VdEG1 and GFP served as controls respectively. Error bars represent standard errors. ** and *** represent significant differences at *P* < 0.01 and *P* < 0.001, respectively, between the native VdSCP76 or VdSCP77 suppression activities and site-directed mutant proteins according to one-way analysis of variance (ANOVA).

**Figure S12**

**
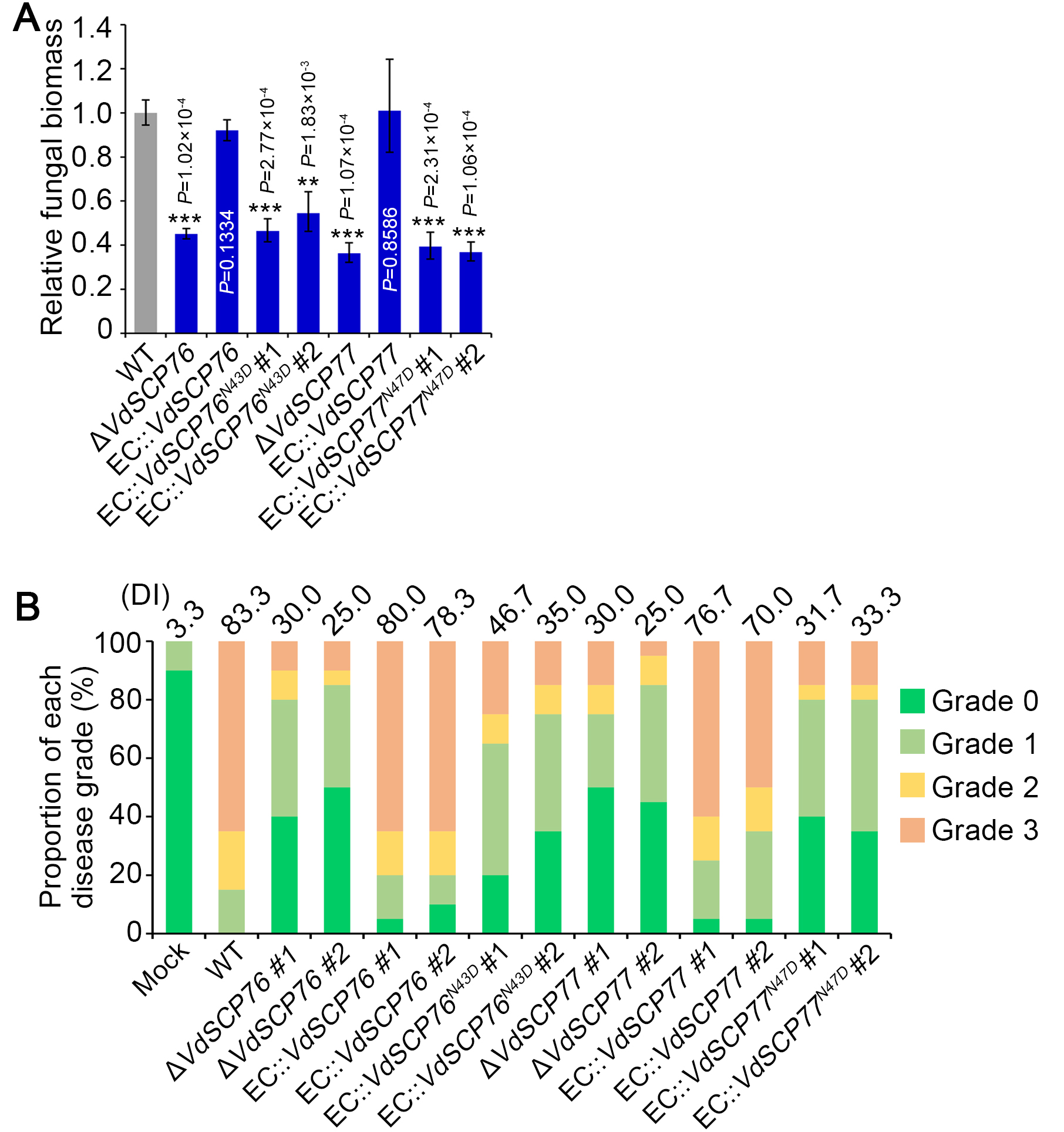
**

**Figure S12. The fungal biomass and disease index of indicated strains on cotton.** (**A**) Three-week-old cotton (cv. Junmian 1) seedlings were inoculated with *VdSCP76* and *VdSCP77* gene deletion, complementary transformants of wild type and site-mutated genes of *VdSCP76* and *VdSCP77*. The fungal biomass of *V. dahliae* on cotton was determined by qPCR at 3 weeks after inoculation. Error bars represent standard errors. ** and *** indicate statistical significance at *P*＜0.01 and *P*＜0.001, respectively, in WT and each knockout mutant or complementary transformant according to one-way analysis of variance (ANOVA). (**B**) The disease was classified as 0–3 scale in which, Grade 0 was 0%–25% leaves wilted, Grade 1 where 25%–50% leaves wilted, Grade 2 where 50%–75% leaves wilted and Grade 3 where 75%–100% leaves wilted. The data were generated from two replicate experiments with a total of 20 cotton seedlings for each *V. dahliae* strain inoculated. The bar chart represents the proportion of each disease grade, and the numbers on top of the column represent the disease indices (DI) calculated from the disease grades. The ratings were conducted with 20 cotton seedlings at 3 wpi with each of the respective *V. dahliae* strains

**Figure S13**


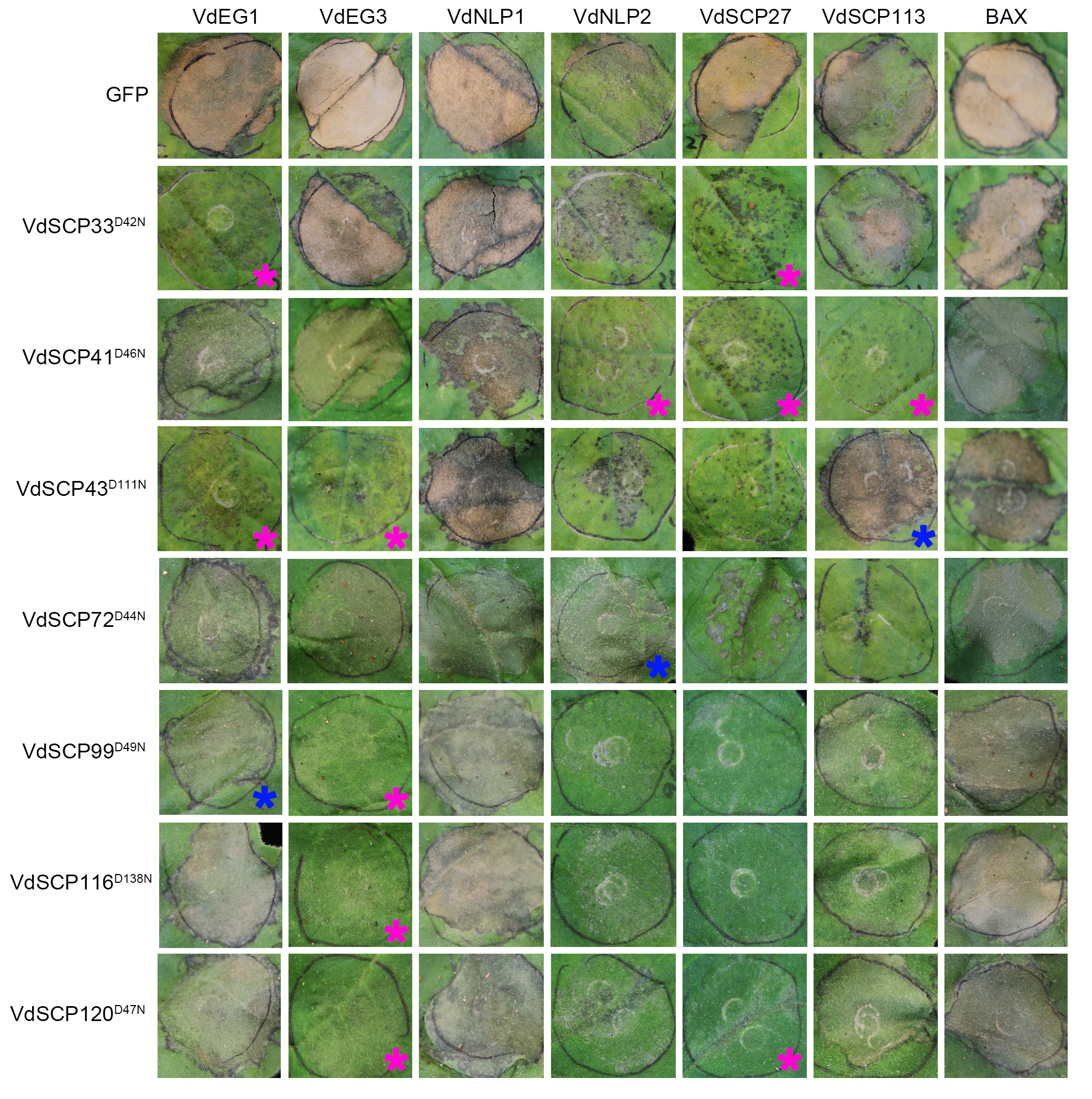


**Figure S13. Functional analyses of conserved asparagine residues in the CFEM domain-containing members from *Verticillium dahliae*.** Cell death suppression activity of site-directed asparagine residue mutant proteins were detected by co-expressing transiently with cell death proteins in 4-week-old *Nicotiana benthamiana* leaves. GFP and cell death proteins co-expression, respectively, were used as controls. The pink and blue asterisks represent the increased- or decreased suppression activity after the site-mutated (D>>N) members respectively.

**Figure S14**


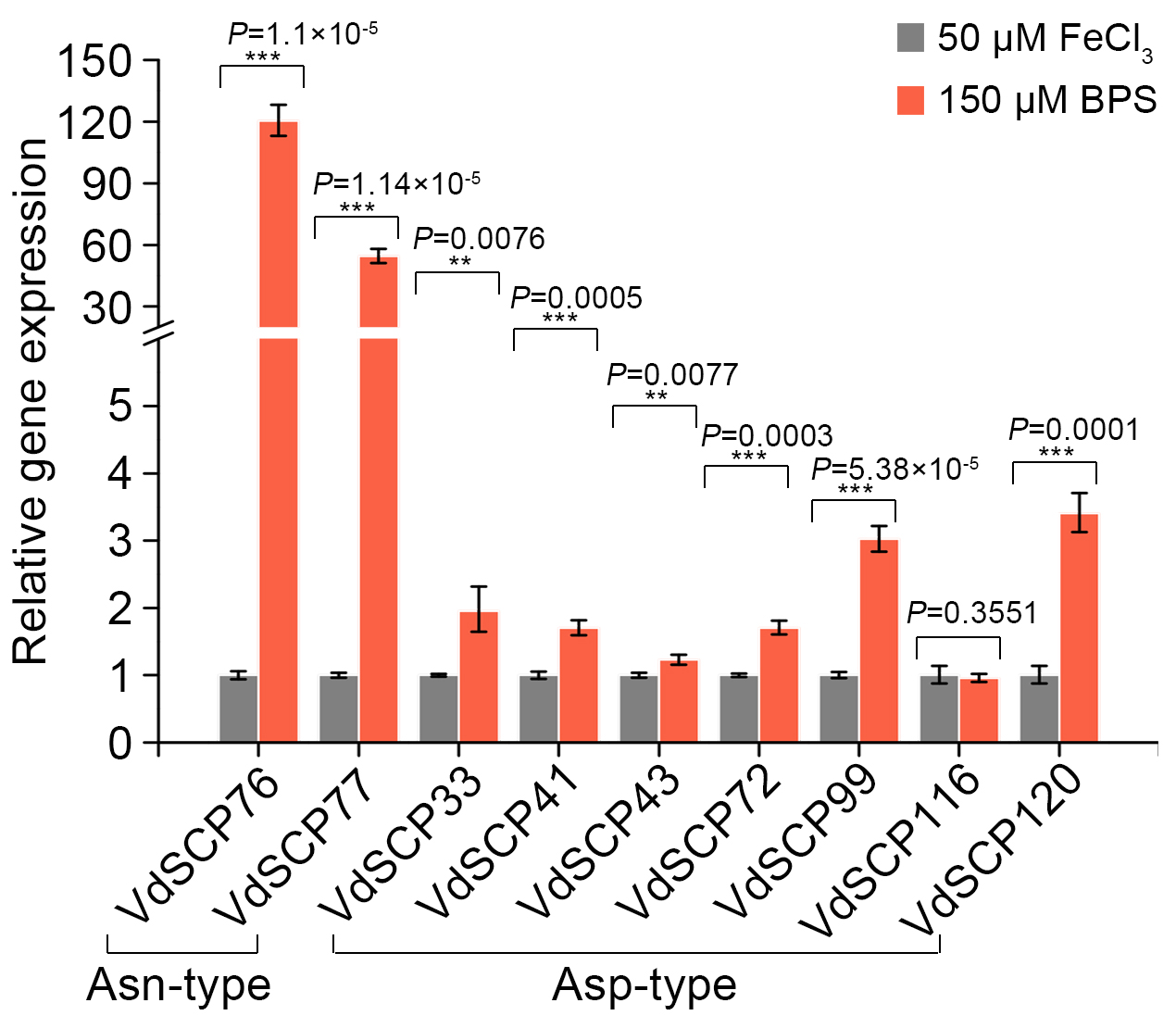


**Figure S14. Gene expression level of CFEM-containing VdSCPs under iron starvation and ferric ion conditions.** Exogenous adding 150 μM bathophenanthrolinedisulfonic acid disodium salt (BPS) 50 μM FeCl_3_ in Czaepk-Dox plates, respectively, were used to create an iron starvation and ferric ion conditions. The *V. dahliae* strain was cultured collected after 3 days of induction. Gene expression level of CFEM-containing VdSCPs family members was detected by RT-qPCR. The *V. dahliae* *EF-1α* was used as endogenous reference.

**Figure S15**


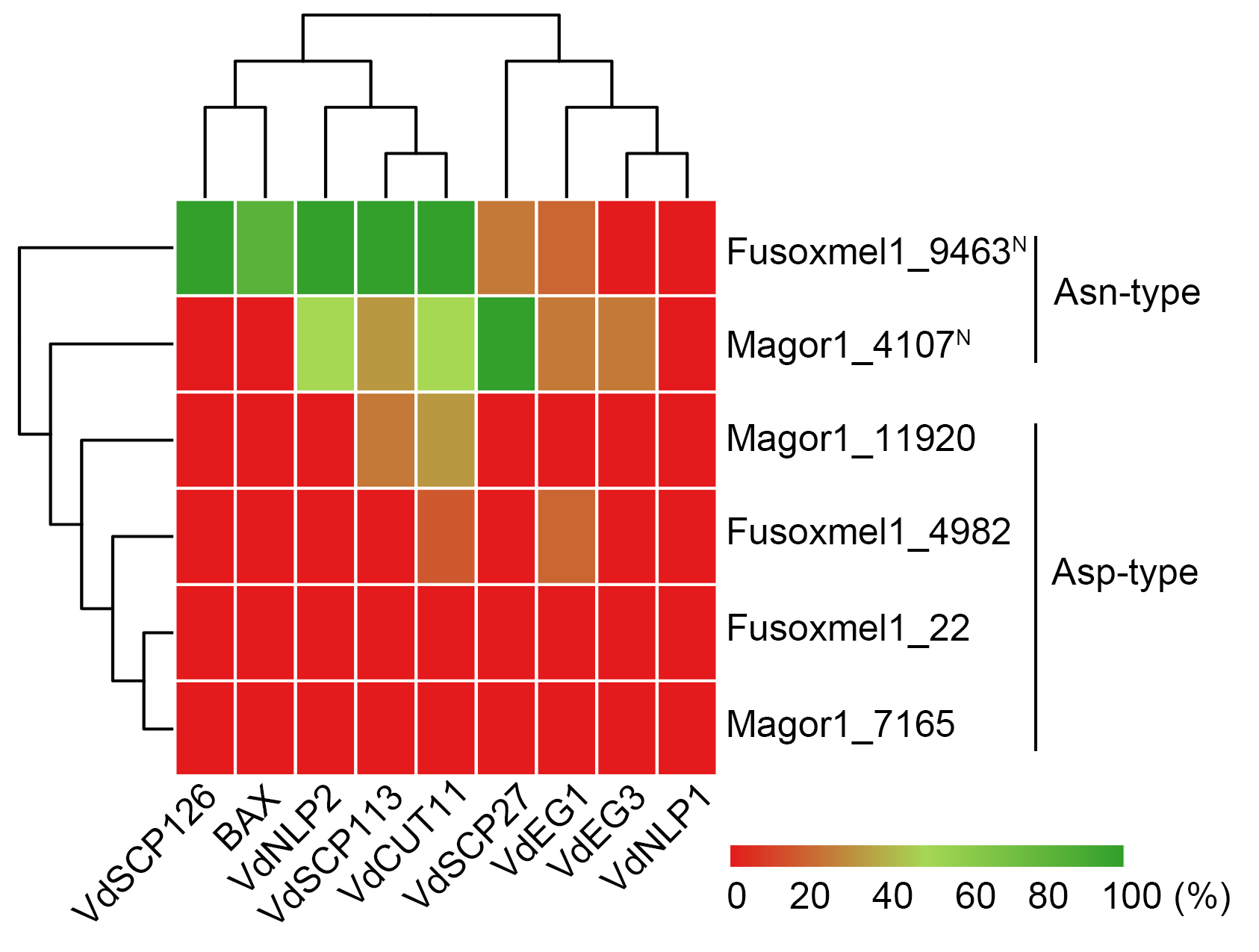


**Figure S15. Functional dissection Asp-type and Asn-type CFEM-containing secretory proteins from *Fusarium oxysporum* and *Magnaporthe oryzae*.**
